# Correlation between secondary metabolites of *Iris confusa* Sealy and* Iris pseudacorus* L. and their newly explored antiprotozoal potentials

**DOI:** 10.1186/s12906-023-04294-0

**Published:** 2023-12-16

**Authors:** Passent M. Abdel-Baki, Moshera M. El-Sherei, Amal E. Khaleel, Essam Abdel-Sattar, Mohamed A. Salem, Mona M. Okba

**Affiliations:** 1 Department of Pharmacognosy, Faculty of Pharmacy, Cairo University, Kasr-El-Ainy Street, Cairo, 11562 Egypt; 2 Department of Pharmacognosy, Faculty of Pharmacy, Menoufia University, Gamal Abd El Nasr St., Shibin Elkom, 32511 Menoufia Egypt

**Keywords:** *Iris confusa*, Antiprotozoal, Antiplasmodial, Antileishmanial, Antitrypanosomal, UPLC-HRMS/MS

## Abstract

**Background:**

In the last few decades, the use of plant extracts and their phytochemicals as candidates for the management of parasitic diseases has increased tremendously. Irises are aromatic and medicinal plants that have long been employed in the treatment of different infectious diseases by traditional healers in many cultures. This study aims to explore the potential of three common *Iris* species (*I. confusa* Sealy*, I. pseudacorus* L. *and I. germanica* L.) against infectious diseases. Their in vitro antiprotozoal potency against *Plasmodium falciparum, Trypanosoma brucei brucei*, *T. b. rhodesiense*, *T. cruzi* and *Leishmania infantum* beside their cytotoxicity on MRC-5 fibroblasts and primary peritoneal murine macrophages were examined.

**Methods:**

The secondary metabolites of the tested extracts were characterized by UPLC-HRMS/MS and Pearsons correlation was used to correlate them with the antiprotozoal activity.

**Results:**

Overall, the non-polar fractions (NPF) showed a significant antiprotozoal activity (score: sc 2 to 5) in contrast to the polar fractions (PF). *I. confusa* NPF was the most active extract against *P. falciparum* [IC_50_ of 1.08 μg/mL, selectivity index (S.I. 26.11) and sc 5] and *L. infantum* (IC_50_ of 12.7 μg/mL, S.I. 2.22 and sc 2). *I. pseudacorus* NPF was the most potent fraction against *T. b. rhodesiense* (IC_50_ of 8.17 μg**/**mL, S.I. 3.67 and sc 3). Monogalactosyldiacylglycerol glycolipid (18:3/18:3), triaceylglycerol (18:2/18:2/18:3), oleic acid, and triterpenoid irridals (spirioiridoconfal C and iso-iridobelamal A) were the top positively correlated metabolites with antiplasmodium and antileishmanial activities of *I. confusa* NPF. Tumulosic acid, ceramide sphingolipids, corosolic, maslinic, moreollic acids, pheophytin *a*, triaceylglycerols, mono- and digalactosyldiacylglycerols, phosphatidylglycerol (22:6/18:3), phosphatidylcholines (18:1/18:2), and triterpenoid irridal iso-iridobelamal A, were highly correlated to *I. pseudacorus* NPF anti-* T. b. rhodesiense* activity. The ADME study revealed proper drug likeness properties for certain highly corelated secondary metabolites.

**Conclusion:**

This study is the sole map correlating *I. confusa* and *I. pseudacorus* secondary metabolites to their newly explored antiprotozoal activity.

**Supplementary Information:**

The online version contains supplementary material available at 10.1186/s12906-023-04294-0.

## Background

The majority of so-called neglected diseases, which disproportionately impact marginalized populations and for whom effective treatments are not readily available due to a variety of reasons, such as high cost, limited compliance, drug resistance, ineffectiveness, and high toxicity, are caused by protozoal infections [1]. Approximately 700,000 people die each year from parasitic diseases, which make up more than 17% of all infectious diseases [2]. Major killers that cause substantial illness and mortality in underdeveloped nations are* Plasmodium* (malaria), *Trypanosoma* (African trypanosomiasis, American trypanosomiasis) and *Leishmania* (leishmaniasis) [3]. The Plasmodium protozoan is responsible for the most common parasite disease in the world, malaria. Malaria is widespread in nearly 100 countries. It caused an estimated 405,000 fatalities and 228 million infections in 2018 [4].

Furthermore, the parasites responsible for African sleeping sickness, also known as Human African Trypanosomiasis (HAT), are the African trypanosomes, (*Trypanosoma brucei rhodesiense* and *Trypanosoma b. gambiense*). Additionally, *Trypanosoma b. brucei* is what causes nagana, or African animal trypanosomiasis, in livestock. Because HAT is fatal if left untreated, sub-Saharan Africa has high rates of morbidity and mortality. It also causes 1.5 million disability-adjusted life years (DALYs), a measure of the loss of 1 year of a healthy and productive life due to disease, which is a financial burden in such regions. The World Health Organisation (WHO) Special Programme for Research and Training in Tropical Diseases (TDR) has so designated HAT as a category 1 disease [5]. Furthermore, over 28 million people are at risk of contracting *T. cruzi*, a protozoan parasite that causes African trypanosomiasis (Chagas disease), which affects 15 million people worldwide [6].

A set of chronic infectious disorders known as leishmaniasis are brought on by the *Leishmania* protozoan [7]. Leshmaniasis is a neglected, resurgent, and uncontrolled tropical disease that affects around 12 million individuals worldwide [8]. Infantile visceral leishmaniasis (Kala-azar) is brought on by *L. infantum* and is prevalent in the Mediterranean region and Latin America [9]. Due to side effects, extended parenteral administration, high cost, low efficacy, and significant drug resistance, the current course of treatment is unfavourable [10].

The rise of emerging trypanosomiasis, leishmaniasis, and malaria in both developing and third-world countries highlight the need for the identification of novel natural effective therapeutic treatments. In order to discover effective, affordable treatments for those lethal parasite diseases, it is therefore required to explore therapeutic plants, extracts, and compounds, known as "hits," that have a specific activity at a non-toxic level [11–13].

In temperate and tropical climates, *Iris* spp., belonging to family Iridaceae, are extensively spread [14]. Irises have been used as effective folk cures for a variety of ailments in many different cultures. *Iris pseudacorus* L. rhizomes were used to treat throat infections in Irish and British traditional medicine [15]. Irises were used in Mongolian traditional medicine, to cure bacterial diseases [16]. Additionaly, irises have been linked to a number of biological activities, including putative anti-bacterial, anti-viral, and antiprotozoal potentials [17–21].

*I. confusa* rhizomes had been used as a folk medicine to treat acute tonsillitis and bronchitis [22]. *I. confusa* whole plant extract showed inhibitory activity on hepatitis B virus (HBV) DNA replication. The isolated compounds, spirioiridoconfal C and 28-deacetyl-belamcandal from *I. confusa* extracts showed potent activities against the HBV DNA replication and did not show inhibitory activities to the secretion of HBsAg and HBeAg [22].

Among the Egyptian cultivated *Iris* species are the three most common and available rhizomatous plants; *I. confusa* Sealy (bamboo iris), *I. pseudacorus* L. (yellow flag), and *I. germanica* L. (German iris) [23, 24] which were chosen for this study. Previously, we studied the anti-virulence; anti-haemolytic and quantitative biofilm inhibition as well as the anti-*Helicobacter pylori* activities of *Iris confusa* Sealy, *I. germanica* L.*,* and *I. pseudacorus* L. cultivated in Egypt [25, 26]. In addition, we investigated their primary [27] and secondary metabolites [25]. In light of our previous work, this study is a continuation of our research regarding the biological potentials of the aforementioned interesting irises against infectious diseases. This study aims to evaluate the in vitro antitrypanosomal (*T. b. brucei*, *T. b. rhodesiense, and T. cruzi*), antiplasmodial (*P. falciparum*-K1), and antileishmanial (*L. infantum*) activities of the polar (PFs) and non-polar (NPFs) fractions of *I. confusa*, *I. pseudacorus,* and *I. germanica* for the first time. In addition, their cytotoxicity on human embryonic lung fibroblasts (MRC-5) and primary peritoneal murine macrophages (PMM) was assessed to evaluate their selectivity, together with their antioxidant activity. This study is the sole map correlating *Iris* secondary metabolites to their newly explored antiprotozoal activity.

## Materials and methods

### General

Inactivated fetal calf serum (FCSi), minimal essential medium (MEM), chlorophenol red *β*-D-galactopyranoside (CPRG), resazurin, nitro blue tetrazolium (NBT), phenazine ethosulfate (PES) and 3-[4,5-dimethylthiazol-2-yl]- 2,5-diphenyltetrazolium bromide (MTT) were purchased from Sigma Aldrich (Bornem, Belgium). Roswell Park Memorial Institute Medium (RPMI-1640) and penicillin–streptomycin (P/S solution) were supplied by Gibco BRL, (Merelbeke,. Belgium). The cell lines; human embryonic lung fibroblasts (MRC-5; Biowhittaker, Verviers, Belgium) and primary peritoneal murine macrophages (PMM (Naval Medical Research Institute (NMRI mouse; Charles River, Sulzfeld, Germany) were used. Standard drugs and all other reagents used were purchased from Sigma Chemical Company (CA, USA).

### Plant material

The flowering *Iris germanica* L. and *Iris pseudacorus* L. (both collected from the Experimental Station of Medicinal Plants of the Faculty of Pharmacy, Assiut University, Egypt) and *Iris confusa* Sealy (from Al-Mansouria, Giza, Egypt), were collected in March 2018. They were graciously authenticated by Prof. Dr. Abd Haleem Abd El-Mogali, chief researcher, Flora and Phytotaxonomy Research Department, Agriculture Museum, Giza, Egypt. The curator of the African Iridaceae at the Royal Botanic Gardens, Kew, in London, UK, Dr. Nina Davies, graciously confirmed and authenticated the identity of the plant sample. Voucher specimens were deposited in the Herbarium of the Pharmacognosy Department at Cairo University with registration number Jan 15, 2019 (I-III).

### Extraction and fractionation

*I. germanica, I. pseudacorus* and* I. confusa* underground parts were separated, air-dried in the shade then powdered. The plant materials were stored in dark containers with tight lids until use. Following the method described in Salem et al., 2016, ten mg of the separated underground parts of each species were individually extracted using a macertation with 1 mL of MTBE: MeOH 3:1 *v/v*. Each sample received an equal volume (3:1 *v/v*) addition of H_2_O and MeOH for liquid–liquid extraction. The lower layers of each sample (H_2_O–MeOH) were evaporated until dryness yielding the polar fractions (PF). The higher layers were evaporated until dryness generating the non-polar fractions (NPFs).

### In vitro antiprotozoal activity

#### Preparation of polar (PFs), non-polar (NPFs) fractions' and reference drugs' stock solutions

Just before screening, the dried plant fractions (PFs and NPFs) and reference drugs were individually dissolved in 100% dimethylsulfoxide (DMSO) at 20 mg/mL and then further diluted with the medium.The DMSO concentration did not exceed 0.5% in order not to affect the parasite growth [28]. To construct a full dose-titration and to determine of the IC_50_ (inhibitory concentration 50%), plant fractions and reference drugs were examined at concentration of 64, 16, 4, 1 and 0.25 μg/mL.

#### Test plate production

Greiner, Bio-One, Wemmel, Belgium was used for the experiments [29, 30]. A robotic station (BIOMEK 2000, Beckman, CA, USA) performed the dilutions. Each plate comprised reference controls (positive control), infected untreated controls (negative control), and blank medium-controls (blanks: 0% growth). All experiments were performed in triplicates (first test in duplicate and one independent repeat).

#### Antiplasmodial potency evaluation

In RPMI-1640 medium supplemented with 25 mM Hepes, 0.37 mM hypoxanthine, 25 mM NaHCO_3_ and ten percent O^+^ human serum together with two percent washed human O^+^ erythrocytes, chloroquine-resistant *Plasmodium falciparum* (K1 strain) was maintained. In 96-well microtiter plates, tests were run in an atmosphere of 3% O_2_, 4% CO_2_ and 93% N_2_. A test material solution containing 10 μL was added to each well along with 190 μL of the malaria parasite inoculum (2% haematocrit, 1% parasitaemia), which was then incubated for 72 h then stored at -20 °C. The Malstat assay, a colorimetric procedure based on the reduction of 3-acetyl pyridine adenine dinucleotide (APAD) by parasite-specific lactate dehydrogenase (pLDH) [31]**,** was used to measure the parasite multiplication after thawing. Twenty μL of each well were transferred into another plate along with 100 μL of the Malstat™ reagent. PES (0.1 mg/mL) and NBT (2 mg/mL) were combined in a volume of 20 μL at a ratio of 1:1. Using a Biorad 3550-UV microplate reader, the colour change (blue formazan product) was measured spectrophotometrically at 655 nm. A dose response curve was used to measure the 50% inhibitory concentration (IC_50_), and the tested samples potency was determined using the aforementioned scoring system.

Score 1: inactive; 2: weak; 3: moderate; 4: pronounced; 5: strong.

#### Antitrypanosomal potency evaluation

*T. b. brucei* (suramin-sensitive, Squib-427 strain) and *T. b. rhodesiense* (STIB-900 strain) trypomastigotes were grown separetly in Hirumi-9 (HMI-9) medium at 5% CO_2_ and 37^◦^C with 10% FCSi. The *T. b. brucei* and *T. b. rhodesiense* assays were carried out as per Vik et al.[1] and Freiburghaus et al. [32] instructions, respectively. Using an excitation λ 536 nm and emission λ 588 nm, The plates of *T. b. rhodesiense* and *T. b. brucei* were read in Molecular Devices Cooperation, CA, USA (Spectramax Gemini XS microplate fluorimeter) [33].

On human lung fibroblast (MRC-5) cells, *Trypanosoma cruzi* Tulahuen CL2 (nifurtimox-sensitive strain) was kept in MEM with 200 mM L-glutamine, 16.5 mM sodium hydrogen carbonate, and 5% FCSi at 37 °C in a 5% CO_2_ atmosphere. After incubation for 7 days at 37°C, 4 × 10^3^ MRC-5 cells and 4 × 10^4^ parasites were introduced to each well. By addition of the *β*-galactosidase substrate CPRG for 4 h at 37 °C, parasite growth was evaluated. The absorbances were represented as a percentage of the blank controls when the colour reaction was measured at 540 nm after 4 h [1].

#### Antileishmanial potency evaluation

Primary peritoneal murine macrophages (PMM) were infected using *L. infantum* (MHOM*/*FR*/*96*/*LEM3323) amastigotes that were extracted from the spleen of a donor hamster that had the infection. Starch was injected intraperitoneally to activate PMM, the host cells used in the experiment. The macrophages were gathered and sown (3 × 10^4^) two days later in each well of a 96-well plate that was being incubated at 37°C with 5% CO_2_. In RPMI-1640 + 5% FCSi, *L. infantum* ex vivo (spleen-derived) amastigotes were employed to infect the PMM at infection ratio 10:1 after two days. The dilutions of the tested samples were added to the plates after a further 2 h of incubation. The plates were then kept at 37°C and 5% CO_2_ for 5 days. After incubation, cells were dried, methanol-fixed, and stained with 20% Giemsa stain for examination. Results were represented as % reduction of amastigote burden (mean number of amastigotes/macrophage) compared with the untreated control cultures (without tested samples) [34].

#### Determination of cytotoxicity and selectivity against MRC-5 and PMM cell lines

By utilising MTT solutions in 96-well microplates, the colorimetric MTT assay was used to determine the cytotoxicity of the examined fractions [35]. MRC-5 and PMM were grown in MEM and in RPMI-1640, respectively supplemented with 20 mM L-glutamine, 5% FCSi and NaHCO_3_ (16.5 mM) at 37^◦^C and 5% CO_2_ and 2% P/S solution. The prediluted sample test plates were seeded with 10^4^cells per well, and they were then incubated for 72 h at 37°C and 5% CO_2_. After incubation, the viability of the cells was assessed using a GENios microplate reader and resazurin. From a dose response curve, the 50% cytotoxic concentration (CC_50_) was calculated. The toxicity of the studied fractions for MRC-5 and PMM as well as their efficacy against the tested parasites were compared using selectivity index (SI). It was computed as follows with regard to the antitrypanosomal and antimalarial action [1]:

$${\text{SIa}}= {{\text{CC}}}_{50} ({\text{MRC}}-5\mathrm{ fibroblasts}) / {{\text{IC}}}_{50} ({\text{parasite}})$$

Concerning the antileishmanial activity, SI was calculated as [1]

$${\text{SIa}}= {{\text{CC}}}_{50} ({\text{MRC}}-5\mathrm{ fibroblasts}) / {{\text{IC}}}_{50} ({\text{parasite}})$$

and [36]

$${\text{SIb}}= {{\text{CC}}}_{50} (\mathrm{PMM macrophages }/ {{\text{IC}}}_{50} ({\text{parasite}})$$

#### UPLC-ESI–MS/MS analysis

This analysis was done according to Okba et al*.* [25]. Statistical analyses were conducted using Pearson’s correlation. Metaboanalyst 3.0 was used for multi-variate data analysis [37].

### Estimation of total phenolic, favonoid and triterpene contents

#### Total phenolic content (TPC) estimation

TPC of PFs and NPFs were calculated using the Folin-Ciocalteu colorimetric method [38]. A standard calibration curve was constructed using gallic acid as a standard. The results were expressed as μg gallic acid equivalent (GAE)/mg dried fraction (DF).

#### Total favonoid content (TFC) estimation

TFC of PFs and NPFs were determined using aluminium chloride method [39]. Quercetin was used as standard. The TFC was calculated from the standard calibration curve and was represented as μg quercetin equivalent (QE) /mg DF.

#### Total triterpene content (TTC) estimation

TTC of PFs and NPFs were determined based on measuring the red–purple color intensity that resulted from perchloric acid-oxidized triterpenes in glacial acetic acid with vanillin reaction [40]. Ursolic acid was used as a standard compound. The TTC was calculated from the standard calibration curve and was expressed as μg ursolic acid equivalent (UAE)/mg DF.

#### Antioxidant potential evaluation

With a few adjustments, the DPPH antioxidant experiment was performed as instructed by Romano et al.[41]. The PFs and NPFs of *I. pseudacorus, I. germanica,* and* I. confusa,* portions were separately dissolved in methanol with the help of sonication to creat serial dilutions. In each instance, the reaction mixture was 200 μL of 0.004% DPPH in methanol and 22 μL of the tested sample. Similar procedures were used to conduct a blank experiment, which used 22 μL of methanol in place of the sample. The DPPH radicals (non-quenched) were assessed at λmax = 492 nm spectrophotometrically.

#### Drug likeness analysis

To explore the properties of the secondary metabolites correlated with the observed activity, Lipinski’s Rule of Five [42] and Veber’s rules [43] were applied. Drug-likeness profiles prediction of the compounds was measured using SwissADME webtool (http://www.swissadme.ch/) [44].

## Results

**Fig. 1:**
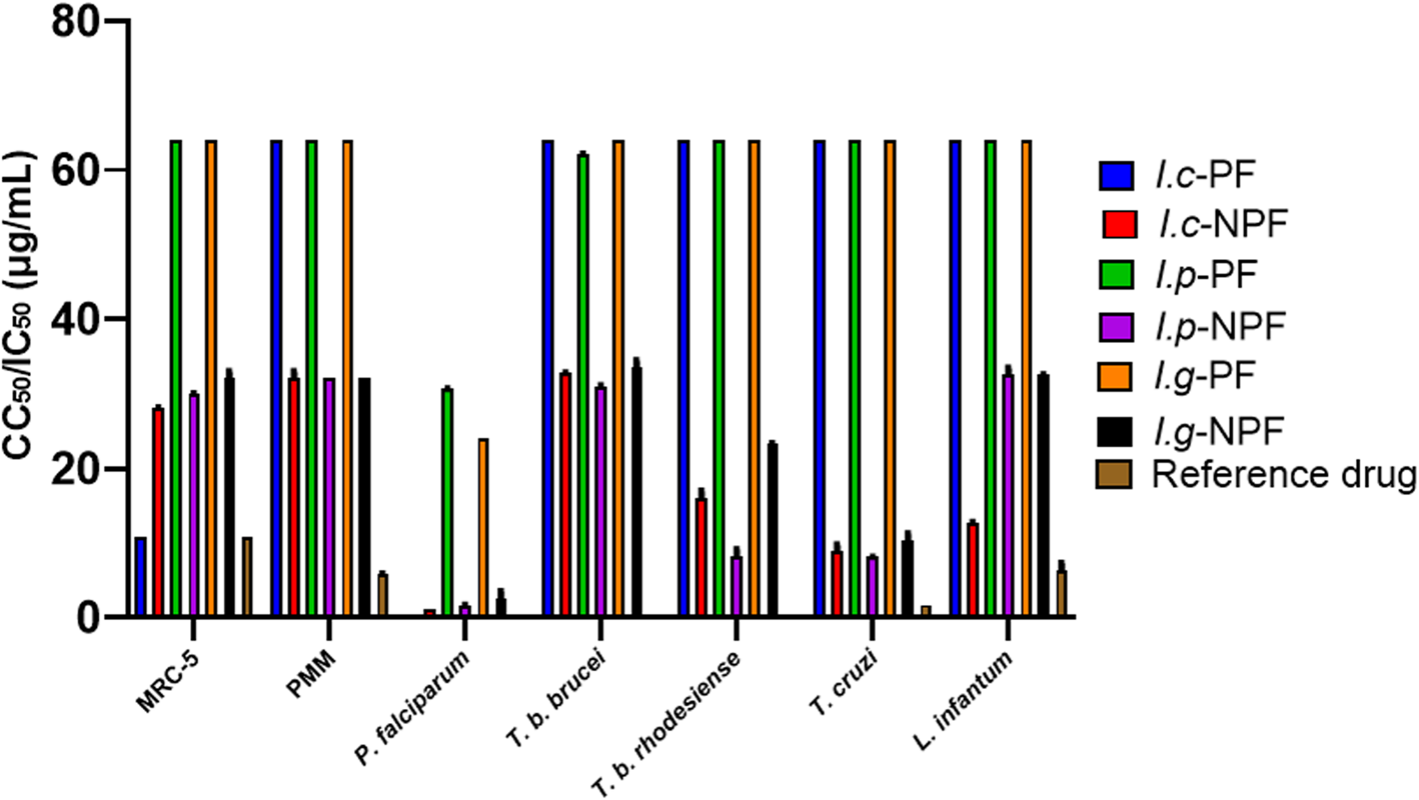
Bar graph representing CC_50_/IC_50_ (μg/mL) of the PFs and NPFs of* I. confusa*, *I. pseudacorus* and *I. germanica* showing their cytotoxicity against MRC-5 and PMM cells as well as their antiprotozoal activity

In a continuation of our interest in exploring plants with antiprotozoal potential [29, 30, 45–47], common *Iris* species from Egypt were screened for their in vitro antiprotozoal potency against *P. falciparum (K-1), T. b. brucei*, *T. b. rhodesiense, T. cruzi*, and *L. infantum.* Their cytotoxicity against PMM and MRC-5 cell lines as well as their selectivity were evaluated (Table 1 and Fig. 1). All NPFs showed potent antiplasmodial, antitrypanosomal and antilishmanial activity than the PFs of the same species.

**Table 1:** Antiprotozoal activity of the PFs and NPFs of *I. confusa*, *I. pseudacorus* and *I. germanica* and their cytotoxicity against MRC-5 and PMM cells

Tested sample			MRC-5		PMM		*P. falciparum*			*T. b. brucei*			*T. b. rhodesiense*			*T. cruzi*			*L. infantum*			
	**μg/mL**		**CC**_**50**_	**Sc**	**CC**_**50**_	**Sc**	**IC**_**50**_	**SI**^**a**^	**Sc**	**IC**_**50**_	**SI**^**a**^	**Sc**	**IC**_**50**_	**SI**^**a**^	**Sc**	**IC**_**50**_	**SI**^**a**^	**Sc**	**IC**_**50**_	**SI**^**a**^	**SI**^**b**^	**Sc**
***I. confusa***		**PF**	10.84^a^	3	> 64.00^a^	1	Nc	Nc	Nc	> 64.00^a^	0.17	1	> 64.00^a^	0.17	1	> 64.00^a^	0.17	1	> 64.00^a^	0.17	0.99	1
		**NPF**	28.15^b^	2	32.00^b^	1	1.08^a^	26.11	5	32.69^b^	0.86	1	16.00^b^	1.76	2	8.83^b^	3.19	3	12.70^c^	2.22	1.00	2
***I. pseudacorus***		**PF**	> 64.00^e^	1	> 64.00^a^	1	30.64^c^	2.09	1	62.14^c^	1.03	1	> 64.00^a^	1.00	1	> 64.00^a^	1.00	1	> 64.00^a^	1.00	5.04	1
		**NPF**	29.96^c^	2	32.00^b^	1	1.61^b^	18.64	4	30.99^d^	0.97	1	8.17^c^	3.67	3	8.14^b^	3.68	3	32.46^b^	0.92	1.00	1
***I. germanica***		**PF**	> 64.00^e^	1	> 64.00^a^	1	23.97^d^	2.67	2	> 64.00^a^	1.00	1	> 64.00^a^	1.00	1	> 64.00^a^	1.00	1	> 64.00^a^	1.00	1.00	1
		**NPF**	32.00^d^	2	32.00^b^	1	2.59^b^	12.34	4	33.45^b^	0.96	1	23.35^d^	1.37	2	10.30^c^	3.11	3	32.46^b^	0.99	0.99	1
**Reference drugs**			10.88^a^		5.80^c^		0.09^a^			0.05^e^			0.05^e^			1.72^d^			6.35^d^			

Scores adopted by Laboratory for Microbiology, Parasitology & Hygiene (LMPH) (μg/mL) for evaluation of the antiprotozoal and cytotoxic activities of the tested fractions. *T. b. brucei,* score 1*:* > 24, 2: > 9, 3: > 3, 4: > 1.2, 5: > 0.4; *T. b. rhodesiense*, score 1: > 24, 2: > 9, 3: > 3, 4: > 1.2, 5: > 0.4; *T. cruzi*, score 1: > 30, 2: > 12, 3: > 2, 4: > 1.51, 5: > 0.55; *L. infantum*, score1: > 30, 2: > 11, 3: > 5, 4:  >1.51  , 5: > 0.5, *P. falciparum* K-1*,* score 1: > 31, 2: > 10, 3: > 4, 4: > 1.5, 5:  > 1.2; cytotoxicity on MRC-5/PMM: no cytotoxicity score 1: > 33, low cytotoxicity score 2: > 11; moderate cytotoxicity score 3: > 5, high cytotoxicity score 4: 1 > 1, 5: > 0.6; Activity score 1: inactive, 2: weak, 3: moderate, 4: pronounced, 5: strong.CC_50_: median cytotoxic concentration; IC_50_: median inhibitory concentration; MRC-5: Diploid human embryonic lung fibroblast; Nc: not completed; NPF: non-polar fraction; PF: polar fraction; PMM: primary peritoneal murine macrophages; SI^a^: selectivity index = CC_50_(MRC-5 fibroblast)/IC_50_(parasite); SI^b^: selectivity index = CC_50_ (PMM macrophages)/IC_50_(parasite); SC: score. IC_50_ and CC_50_ in μg/mL. Reference drugs; Tamoxifen for MRC-5, Amphotercin B for PMM, Chloroquine for *P. falciparum,* Suramin for *T. b. brucei and T. b. rhodesiense,* Benznidazole for *T. cruzi,* Miltefosine for *L. infantum.* Different letters in each column indicate significant differences at *P* < 0.0001 with Tukey’s test

### Cytotoxicity and selectivity

All the tested fractions were non cytotoxic (sc 1) or showing low cytotoxicity (sc 2) against MRC-5 cells except *I. confusa* PF which showed moderate cytotoxicity (sc 3). The PF of *I. confusa* was not further tested because of its high cytotoxicity. All the tested fractions were non cytotoxic against PMM cells (sc 1). The potentially active tested fractions demonstrated nonspecific activity towards certain species of protozoa.

### Antiplasmodial activity

The NPFs of the three *Iris* species displayed pronounced strong activity (sc 4–5) with IC_50_ values in the range 1.08 – 2.59 μg/mL and SI^a^ ratio [CC_50_ (MRC-5 fibroblasts) / IC_50_ (parasite)] in the range 26.11 – 12.34. On the other hand, the PFs showed weak to no activity (sc 1–2). The most potent fraction against* P. falciparum* was* I. confusa* NPF (IC_50_ of 1.08 μg/ml, S.I. 26.11 and sc 5).

### Antitrypanosomal activity

The NPFs of *I. pseudacorus, I. germanica* and *I. confusa* showed weak to moderate activity (sc 2–3) against *T. b. rhodesiense* and *T. cruzi,* while the PFs exerted no activity against the three tested *Trypanosoma* species. *I. pseudacorus* NPF was the most potent fraction against *T. b. rhodesiense* (IC_50_ of 8.17 μg**/**ml, S.I. 3.67 and score 3). The three *Iris* species exerted moderate activity (sc 3) against *T. cruzi* with an IC_50_ of 8.14–10.30 μg**/**ml. On the other hand, *T. b. brucei* was resistant to all fractions.

### Antileishmanial activity

In addition to testing the cytotoxicity of the tested fractions on MRC-5, it was also determined on the primary peritoneal murine macrophages (PMM), the host cells for the amastigote form of *Leishmania* [36]. The selectivity index (SI^b^ = CC_50_ for macrophage / IC_50_ for amastigotes) was used to compare the toxicity of the tested fractions for PMM and the activity against amastigotes of *Leishmania* [36]. Only the NPF of *I.confusa* exerted weak activity (sc 2) against *L. infantum* with IC_50_ of 12.70μg*/*mL. It showed no cytotoxicity (sc 1) on PMM.

### UPLC-ESI–MS/MS analysis

Secondary metabolites profiling of the NPFs of *I. pseudacorus, I. germanica,* and *I. confusa* revealed the presence of 45 metabolites belonging to differenet chemical classes; triterpene acids, iridals, caged-tetraprenylated xanthone, phosphatidic acids, fatty acids, phosphatidyl glycyerols, glycolipids, phosphatidyl ethanol amine, chlorophyll derivatives, phosphatidylcholines, triacylglycerols and ceramides [25]. Pearson’s correlation was applied on this results to explore the top correlated metabolites with the newly observed plasmodicidal, lesishmanicidal and trypanocidal potential.

**Fig. 2:**
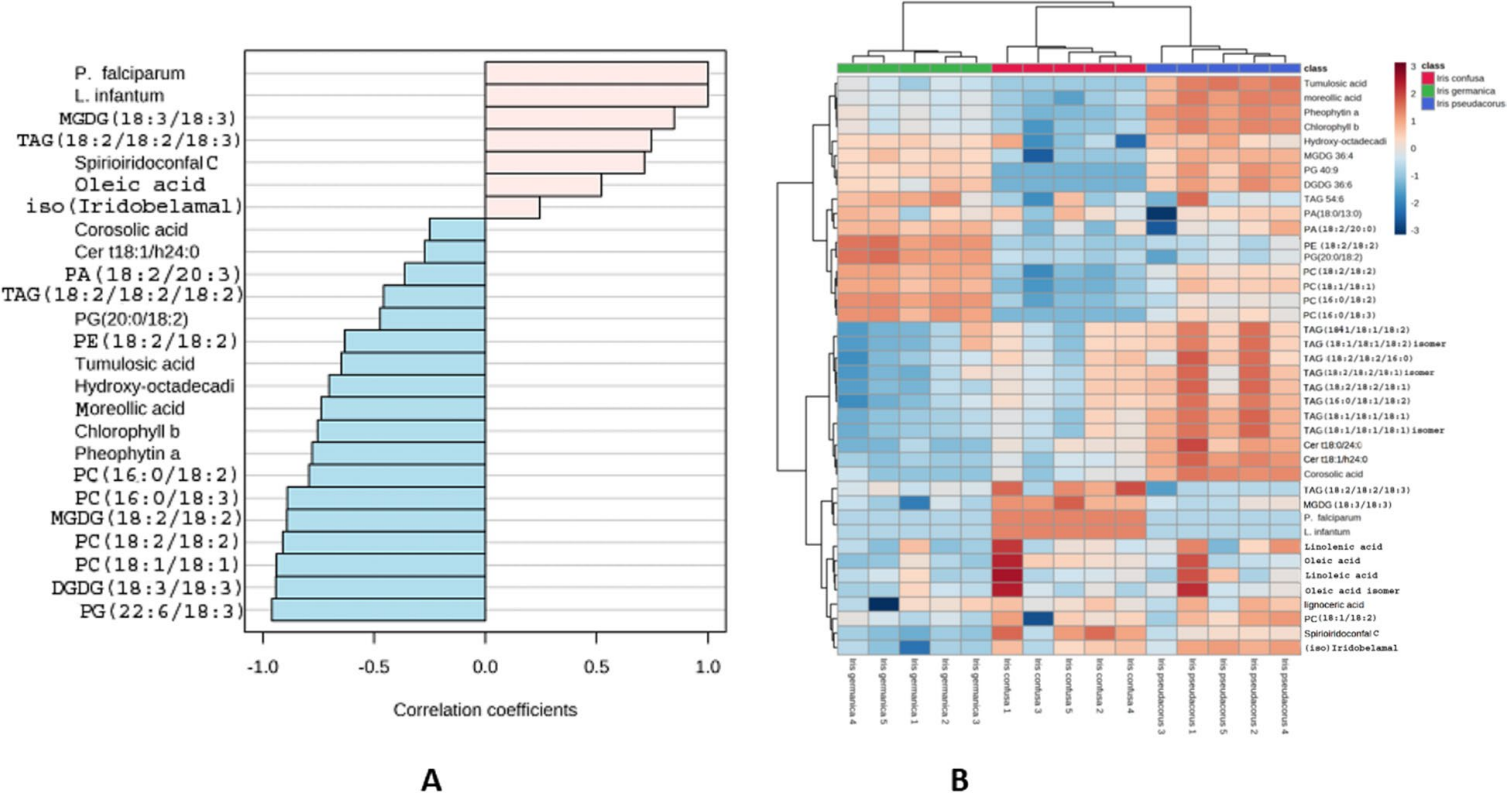
Top *I. confusa* NPF metabolites correlated with its antiplasmodium and antileshmanial activities. A: Pearson’s correlation coefficients indicate the relationship between metabolites and activity against *P. falciparum* and *L. infantum*. B: Heat map for the distribution of metabolites correlated with *I. confusa* NPF activity against *P. falciparum* and *L. infantum*. The metabolite abundance from five biological replicates was used for the generation of heat maps

Interestingly, five metabolites were strongly correlated with *I. confusa* NPF high antiplasmodial and antileshmanial potentials (Fig. 2A and B). These compounds belong to various chemical classes viz glycolipids monogalactosyldiacylglycerol MGDG (18:3/18:3) (metabolite no. 1), triacyl glycerols TAG (18:2/18:2/18:3) metabolite no. 2, iridals (spirioiridoconfal C and, (iso)iridobelamal metabolites no. 3 and 4, respectively, and fatty acid; oleic acid metabolites no. 5.

**Fig. 3:**
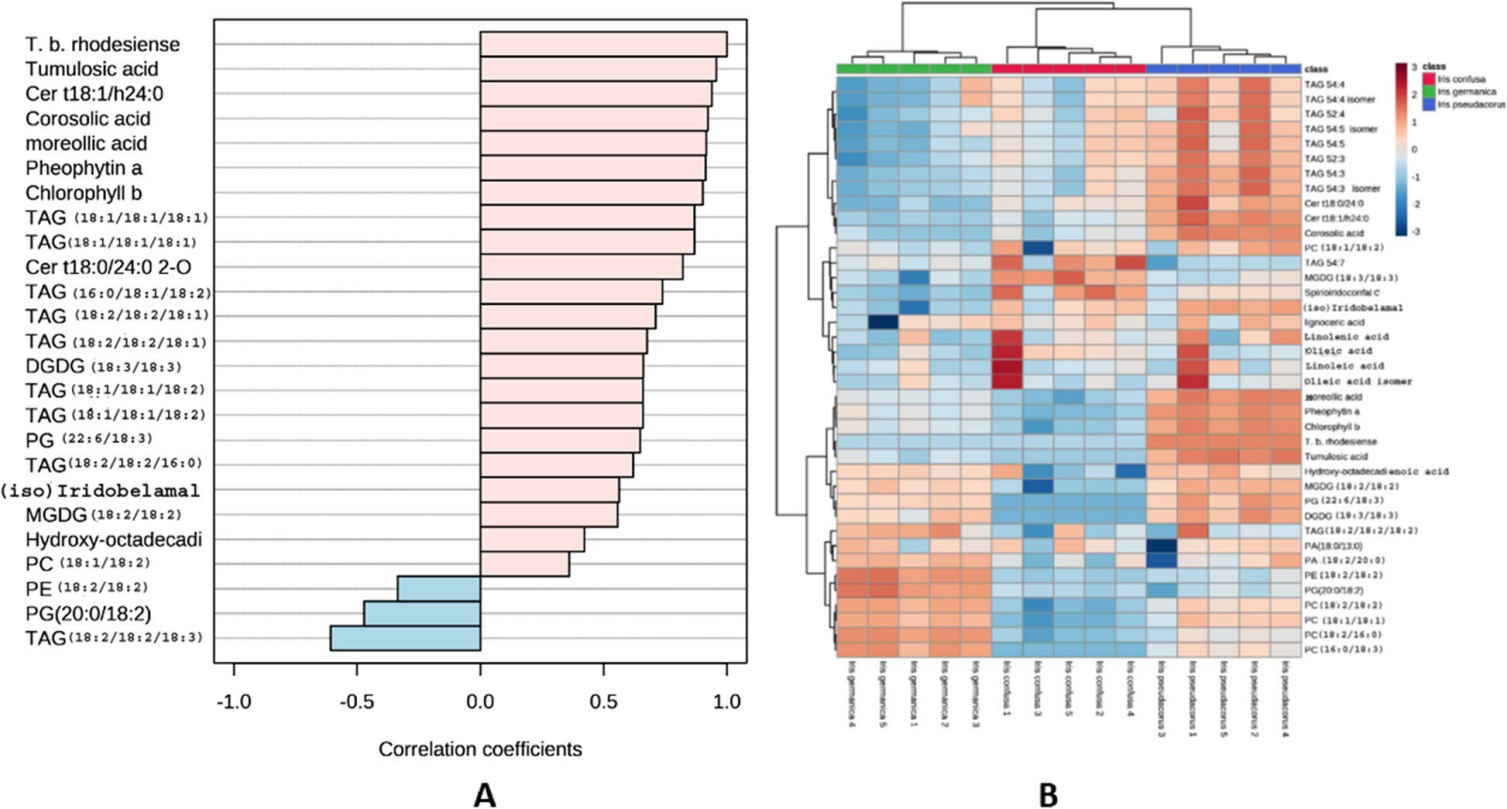
Top *I. pseudacorus* NPF metabolites correlated with its antitrypanosomal activity. A) Pearson’s correlation coefficients indicate the relationship between metabolites and activity against *T. b. rhodesiense*, B) Heat map for the distribution of metabolites correlated with *I. pseudacorus* NPF activity against *T. b. rhodesiense.* The metabolite abundance from five biological replicates was used for the generation of heat maps

On the other hand, thirteen metabolites belonging to different phytochemical classes were highly correlated to *I. pseudacorus* NPF anti-* Trypanosoma b. rhodesiense* activity (Fig. 3A and B) including triterpene acids; tumlosic acid metabolite no. 6 and corosolic/maslinic acid metabolite no. 7, ceramide (t18:1/*α*24:0) metabolite no. 8 and ceramide (t 18:0/*α*24:0) metabolite no. 9, caged xanthone; moreollic acid metabolite no. 10, chlorophyll derivatives: pheophytin a metabolite no. 11 and chlorophyll b metabolite no. 12, phosphatidylglycerol (22:6/18:3) metabolite no. 13, glycolipids: digalactosyldiacylglycerol DGDG (18:3/18:3) metabolite no. 14 and MGDG 18:2/18:2 metabolite no. 15, triterpenoid irridal; iso(iridobelamal) metabolite no. 16, fatty acid; hydroxyoctadecadienoic acid metabolite no. 17, and phosphatidylcholine PC 18:1/18:2 metabolite no. 18.

The structures of the secondary metabolites strongly correlated with* I. confusa* NPF antiplasmodial and antileshmanial potentials and *I. pseudacorus* observed anti* Trypanosoma brucei* rhodesiense potential were represented in Fig. 4 and their Ms/Ms fragmentation were demonstrated in supplementry file (Figs. S1-S18).

**Fig. 4:**
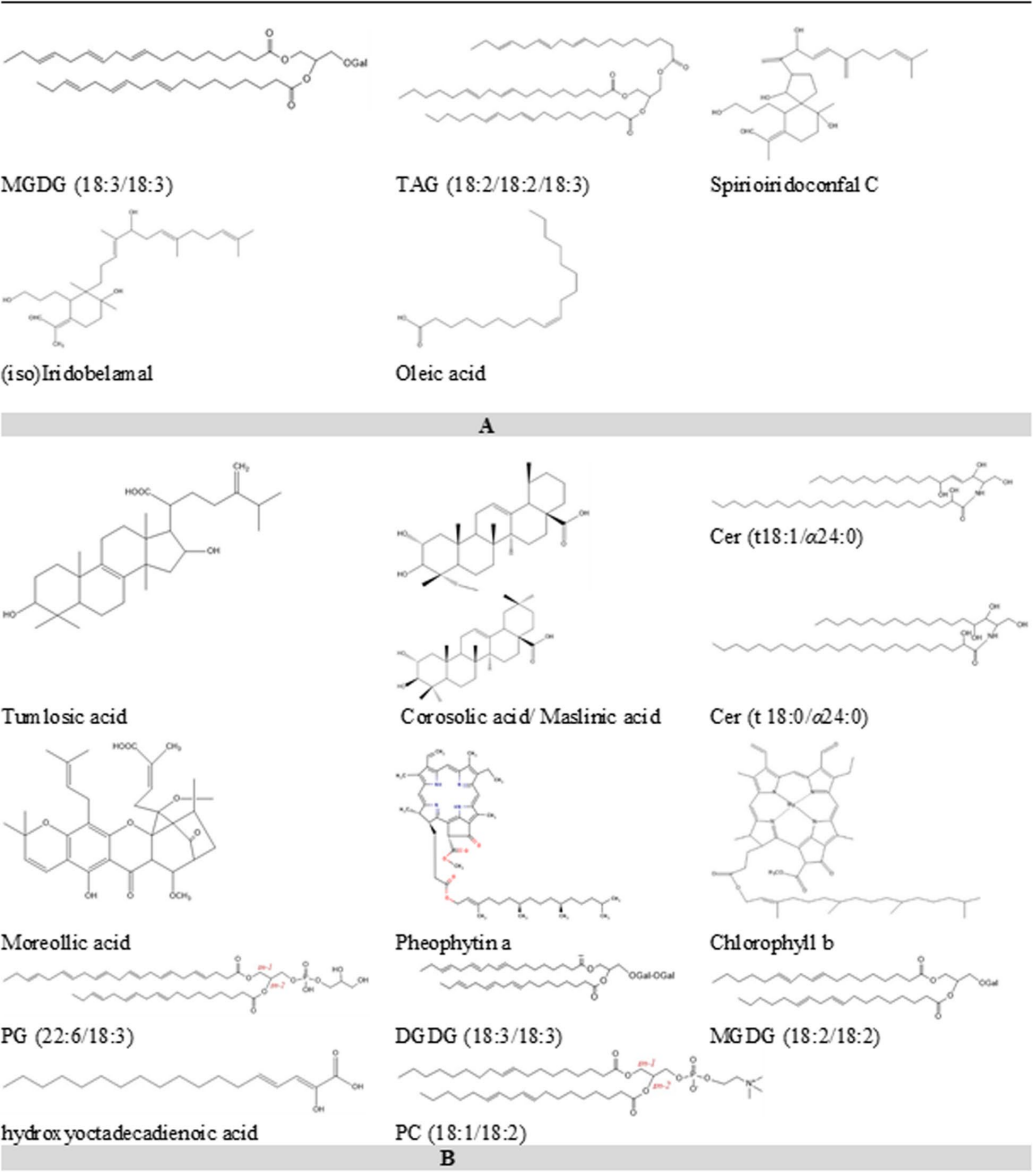
Structures of the secondary metabolites strongly correlated with A)* I. confusa* NPF antiplasmodial and antileshmanial potentials and B)* I. pseudacorus* observed anti* Trypanosoma brucei* *rhodesiense* potential

### Identification of *I. confusa* NPF secondary metabolites that were strongly correlated with the high antiplasmodial and antileshmanial potentials

The following is the detailed MS/MS fragmentation explanation aided in the identification of the highly correlated metabolities recently identified in our previous study [25] on the same common *Iris* species.

**Metabolite no. 1:** It was detected as [M + NH_4_]^+^ ions in the positive mode and as [M + CH_3_COO]^−^ in the negative mode as well documented [48, 49]. Its ammonium adduct showed loss of NH_3_ plus loss of galactosyl residue (-179 Da) with formation of the daughter ion [M + NH_4_-NH_3_-Gal]^+^ at *m/z* 613.48 [48]. Moreover, the ammonium adduct exhibited neutral loss of NH_3_ and loss of galactosyl residue involving the cleavage of the sugar hemiacetal with proton transfer (-197 Da) with formation of daughter ion at *m/z* 595.47. In the positive ionization mode, product ion [RCO + 74]^+^ was observed at *m/z* 335.26 (18:3 FA) which identified the fatty acyl substituents; corresponding to the acyl ion with additional 74 amu equivalent to C_3_H_6_O_2_ (glyceryl moiety) [50]**.** In the negative ionization mode, its acetate adduct yielded the respective fatty acid carboxy anion [C18:3-H]^−^ at *m/z* 277.22 at positions *sn*-1 and *sn*-2 of the glycerol backbone [48]. The acetate adduct of metabolite no. 1 yielded the respective fatty acid carboxy anions [C18:3-H]^−^ at *m/z* 277.22 at positions *sn*-1 and *sn*-2 of the glycerol backbone [48]. It was identified as MGDG (18:3/18:3) (Fig. S1).

**Metabolite no. 2** was detected in positive ionization mode only. A protonated molecular ion at *m/z* 877.72 [M + H]^+^ was observed. Fragment ion appeared at *m/z* 597.49 corresponding to diacyl product ion due to the loss of neutral C18:2 C_17_H_31_COOH from the protonated molecular ion [M + H]^+^ or the loss of 297 Da relative to the loss of neutral C18:2 C_17_H_31_COONH_4_ from the ammonium adduct. Other less abundant fragment ions appeared which were very important in stereoisomers assignments corresponding to C18:2 (*m/z* 263.24, 245.24, 337.27 and 319.26) and others corresponding to 18:3 fatty acid were detected (*m/z* 261.22, 243.21, 335.26 and 317.25). The product ion at *m/z* 599.50 corresponded to the loss of 18:3 fatty acid. The higher abundance of the DAG ion produced by the loss of 18:2 than the one produced from the loss of fatty acid 18:3 suggested that 18:2 and 18:3 acids were located at *sn-1* and *sn-3* positions, respectively. Fragment ion appeared at *m/z* 261.22 [C_14_H_25_CH_2_CH = CH-CO]^+^ corresponding to the typical loss of the middle fatty acid at the *sn*-*2* position as *α*,*β*-unsaturated acid [C_18_H_30_O_2_ + H]^+^ followed by loss H_2_O molecule corresponding to C18:2 at *sn-2*. This was further confirmed by the higher abundance of the product ion at *m/z* 337.27 corresponding to 18:2 [C_17_H_31_CO + 74]^+^ than the ion at *m/z* 335.26 corresponding to 18:3 [C_17_H_29_CO + 74]^+^ showing that the 18:2 fatty acid not 18:3 was located at the *sn-2* position. It was identified as triacyl glycerol (18:2/18:2/18:3) (Fig. S2).

**Metabolites no. 3 and 4:** in the positive ionization mode, fragment ions appeared at *m/z* 469.33/457.37 [M + H-H_2_O]^+^ and 451.32/439.36 [M + H-2H_2_O]^+^ due to loss of 2 successive water molecules [51] from the two compounds, respectively. In the negative ionization mode of (iso)iridobelamal (metabolite no. 4), fragment ion appeared at *m/z* 455.36 due to loss of water molecule. While spirioiridoconfal C (metabolite no. 3) exhibited successive loss of 2 water molecules leading to fragment ions at *m/z* 467.32 and 449.30 [22, 52, 53]. They were identified as spirioiridoconfal C and (iso)iridobelamal irridal respectively (Figs. S3 and S4).

**Metabolites no. 5:** The most abundant ion for the fatty acid is the molecular ion peak [M-H]^−^ only at *m/z* 281.24 [54]. It was identified as oleic acid (Fig. S5).

### Identification of *I. pseudacorus* secondary metabolites that were strongly correlated with the observed anti *T. b. rhodesiense *potential

**Metabolites no. 6 and 7**: they were characterized by fragment ion [M-H-CH_2_O-H_2_O]^−^ at *m/z* 437.34 and 423.33, respectively due to loss of formaldehyde and water molecule (48 Da). [55, 56]**.** They were identified as tumlosic and corosolic/maslinic triterpene acids respectively (Figs. S6 and S7).

**Metabolites no. 8 and 9:** The protonated pseudomolecular molecular ion [M + H]^+^ of metabolite no. 8 yielded daughter ions at *m/z* 664.62 [M + H-H_2_O]^+^ and 646.62 [M + H-2H_2_O]^+^. Fragment ions at *m/z* 298.27, 280.26 and 262.25 were assigned to 6-hydroxysphing-4-enine moiety formed after amide bond cleavage and concomitant loss of three consecutive water molecules. On the other hand, the protonated pseudomolecular ion [M + H]^+^ of metabolite no. 9 yielded daughter ions at *m/z* 666.64 [M + H-H_2_O]^+^, 648.63 [M + H-2H_2_O]^+^and 630.63 [M + H-3H_2_O]^+^. In the positive ionization mode; fragment ion at *m*/*z* 318.30 [C_18_H_39_NO_3_ + H]^+^ assigned to phytosphingosine moiety formed after amide bond cleavage was detected. Abundant triplet fragment ions were detected at *m/z* 300.29, 282.28 and 264.27 formed by subsequent loss of water from the phytosphingosine [57]. Regarding the negative ionization mode of metabolites no. 8 and 9, fragment ions appeared at *m/z* 662.61, and 664.62 [M-H-H_2_O]^−^ and 644.60 and 646.62 [M-H-2H_2_O]^−^, respectively. In addition, fragment ion was detected in the negative ionization of metabolite no. 9 at *m/z* 652.63 [M-H-HCHO]^−^. Moreover prominent fragment ion at *m/z* 383.35 [C_24_H_47_O_3_]^−^ was detected corresponding to 2-hydroxy tetraeicosanoic ion in the spectra of both metabolites. Other fragment ions were observed at *m/*z 365.34 and [RCO_2_^−^-H_2_O] and 337.35 (RCO_2_^−^-[H_2_ + CO_2_]) which were characteristic to the *α*h24:0-fatty acid [58] (Figs. S8 and S9).

In metabolite no. 8, the cleavage of the C2-C3 bond of the LCB led to the formation of N-acylethanolamine (NAE) anion ([NAE–H]^−^) at *m/z* 426.40 [C_23_H_47_CO_2_NHCH_2_CH_2_O]^−^ reflecting fatty acyl substituent to be C24:0 (2OH). Fragment ions reflecting the 6-hydroxysphing-4-enine LCB were also seen at *m/z* 314.27 [LCB-H]^−^ = [C_18_H_34_O_3_NH_3_-H]^−^, 279.23 [M-H-H_2_O-C_23_H_47_CO_2_NH_2_]^−^ (elimination of the fatty acyl moiety as an amide), 253.22 [M-H-NAE]^−^ = [M-H-C_23_H_47_CO_2_NHCH_2_CH_2_OH]^−^.

The ions at *m/z* 426.40 [NAE-H]^−^ = [C_23_H_47_CO_2_NHCH_2_CH_2_OH-H]^−^, 424.38 [NAE-H-2H]^−^ = [C_23_H_47_CO_2_NHCH_2_CH_2_OH-H-2H]^−^, 408.39 [NAE-H-H_2_O]^−^ = [C_23_H_47_CO_2_NHCH_2_CH_2_OH-H-H_2_O]^−^, 383.35 [RCO_2_]^−^ = [C_23_H_47_CO_3_]^−^, and 382.37 [RCONH]^−^ = [C_23_H_47_CO_2_NH]^−^ indicating the h24:0 fatty acyl substituent were prominent. The ions of *m/z* 383.35 along with ions of *m/z* 365.34 [383.35–H_2_O]-, and 337.35 [383.35–(H_2_ + CO_2_)], suggested the presence of *α*h24:0-fatty acyl substituent [58].

Considering metabolite no. 9, the cleavage of the C2-C3 bond of the LCB led to the formation of N-acylethanolamine (NAE) anion ([NAE-H]^−^) at *m/z* 426.40 [C_23_H_47_CO_2_NHCH_2_CH_2_O]^−^ reflecting fatty acyl substituent to be C24:0(2OH). Ions characteristic for t18:0-LCB (phytosphigosine) component were detected at *m/z* 267.23 and 255.23 [58].

Metabolites no. 8 and 9 were identified as ceramide (t18:1/*α*24:0) and ceramide (t 18:0/*α*24:0), respectively.

**Metabolite no. 10**: showed a pseudomolecular ion in the positive ionization mode at *m/z* 593.28 [M + H]^+^ [59]**.** It was identified as caged xanthone (moreollic acid) (Fig. S10).

**Metabolites no. 11 and 12**: The most abundant fragment ions in chlorophyll derivatives is corresponded to the loss of groups from the C-17 position in the form phytil chain (as the phytadiene, C_20_H_38_) or CH_3_COOC_20_H_39_ group [60]. The protonated pseudomolecular ions of metabolite no. 11 yielded product ion at *m/z* 593.27 corresponding to [M + H-C_20_H_38_]^+^. Product ions at *m/z* 533.25 [M + H-CH_3_COOC_20_H_39_]^+^ and other at *m/z* 812.55 [M + H-COOCH_3_]^+^ were detected due to the loss of COOCH_3_ from position C-13_2_ [60]. It was identified as pheophytin a (Fig. S11). The protonated pseudomolecular ion of metabolite no. 12 yielded product ions at *m/z* 629.23 [M + H-C_20_H_38_]^+^ and 569.20 [M + H-CH_3_COOC_20_H_39_]^+^. In addition, fragment ion at *m/z* 627.21 [M-H-C_20_H_38_]^−^ was detected [61]. It was identified as chlorophyll b (Fig. S12).

**Metabolite no. 13:** Carboxylate anions appeared at *m/z* 327.22 [C_21_H_31_CO_2_]^−^ and 277.22 [C_17_H_29_CO_2_]^−^ relative to 22:6 and 18:3 fatty acids. Their relative intensities indicated the C-22:6 is in position *sn-1* (less abundant peak at *m/*z 327.22) while C-18:3 is in position *sn-2* (more abundant peak at *m/z* 277.22). Fragment ions detected at *m/z* 537.28 [M-H-C_18_H_30_O_2_]^−^ and at *m/z* 487.25 [M-H-C_22_H_32_O_2_]^−^ corresponding to the loss of C-18:3 and C-22:6 fatty acids. It was identified as phosphatidylglycerol (22:6/18:3) (Fig. S13).

**Metabolites no. 14 and 15:** were detected as [M + NH_4_]^+^ ions in the positive mode and as [M + CH_3_COO]^−^ in the negative mode as well documented [48]. Their ammonium adduct showed loss NH_3_ plus loss of galactosyl residue (-179 Da) with formation of the daughter ion [M + NH_4_-NH_3_-Gal]^+^ at *m/z* 775.54 and 617.51, respectively [48]. Moreover, the ammonium adduct exhibited neutral loss of NH_3_ plus loss of galactosyl residue involving the cleavage of the sugar hemiacetal with proton transfer (-197 Da) with formation of daughter ion at *m/z* 757.52 and 599.50, respectively. In the positive ionization mode of metabolite no. 14 and, product ion [RCO + 74]^+^ was observed at *m/z* 335.26 (18:3 FA) and 337.27 (18:2 FA) which identified the fatty acyl substituents; corresponding to the acyl ion with additional 74 amu equivalent to C_3_H_6_O_2_ (glyceryl moiety), in each compound respectively [49, 50]. Also, the ammonium adduct of metabolite no. 14 underwent loss of 2 hexose residues and NH_3_ (-341 Da) yielding fragment ion at *m/z* 613.48 [M + NH_4_-2Hex-NH_3_]^+^ was observed. Fragment ion at *m/z* 261.22 [RCO]^+^  = [C_17_H_29_CO]^+^ was detected. In the negative ionization mode, the acetate adduct of metabolites no. 14 and 15 yielded the respective fatty acid carboxy anions [C18:3-H]^−^ at *m/z* 277.22 and [C18:2-H]^−^ at *m/z* 279.23, respectively at positions *sn*-1 and *sn*-2 of the glycerol backbone [48]. The acetate adduct of metabolites no. 14 and 15 yielded the respective fatty acid carboxy anions [C18:3-H]^−^ at *m/z* 277.22 and [C18:2-H]^−^ at *m/z* 279.23, respectively at positions *sn*-1 and *sn*-2 of the glycerol backbone [48]**.** Metabolites no. 14 and 15 were identified as DGDG (18:3/18:3) and MGDG 18:2/18:2) glycolipids (Figs. S14 and S15).

**Metabolite no. 16:** as described above for metabolite no. 3 (Fig. S16).

**Metabolite no. 17:** The most abundant ion for fatty acids is the molecular ion peak [M-H]^−^ only [54]. It was identified as hydroxyoctadecadienoic (FA 16:0) fatty acid (Fig. S17).

**Metabolite no. 18:** it showed fragment ions at *m/z* 279.23 [C18:2-H]^−^ and 281.25 [C18:1–H]^–^ correspond to linoleic and oleic acids, respectively. Small signals detected at *m/z* 506.33 and 504.31 due to loss of ketenes of linoleic [M-CH_3_-262]^−^ and oleic [M-CH_3_-264]^−^ acids, respectively, were observed. Loss of neutral linolenic and oleic acids from the demethylated molecular ion [M-CH_3_]^−^ was found with low intensity at *m/z* 488.32 [M-CH_3_-C18:2]^−^ and 486.30 [M-CH_3_-C18:1]^−^. Product ion relative to the carboxylate anion of linoleic acid at *m/z* 279.23 was more abundant than that relative to oleic acid with more abundance of the ketene of linolenic than that of oleic acid. It was identified as phosphatidylcholine (PC 18:1/18:2) (Fig. S18).

### Quantitative determination of the main phytochemical classes

**Fig. 5:**
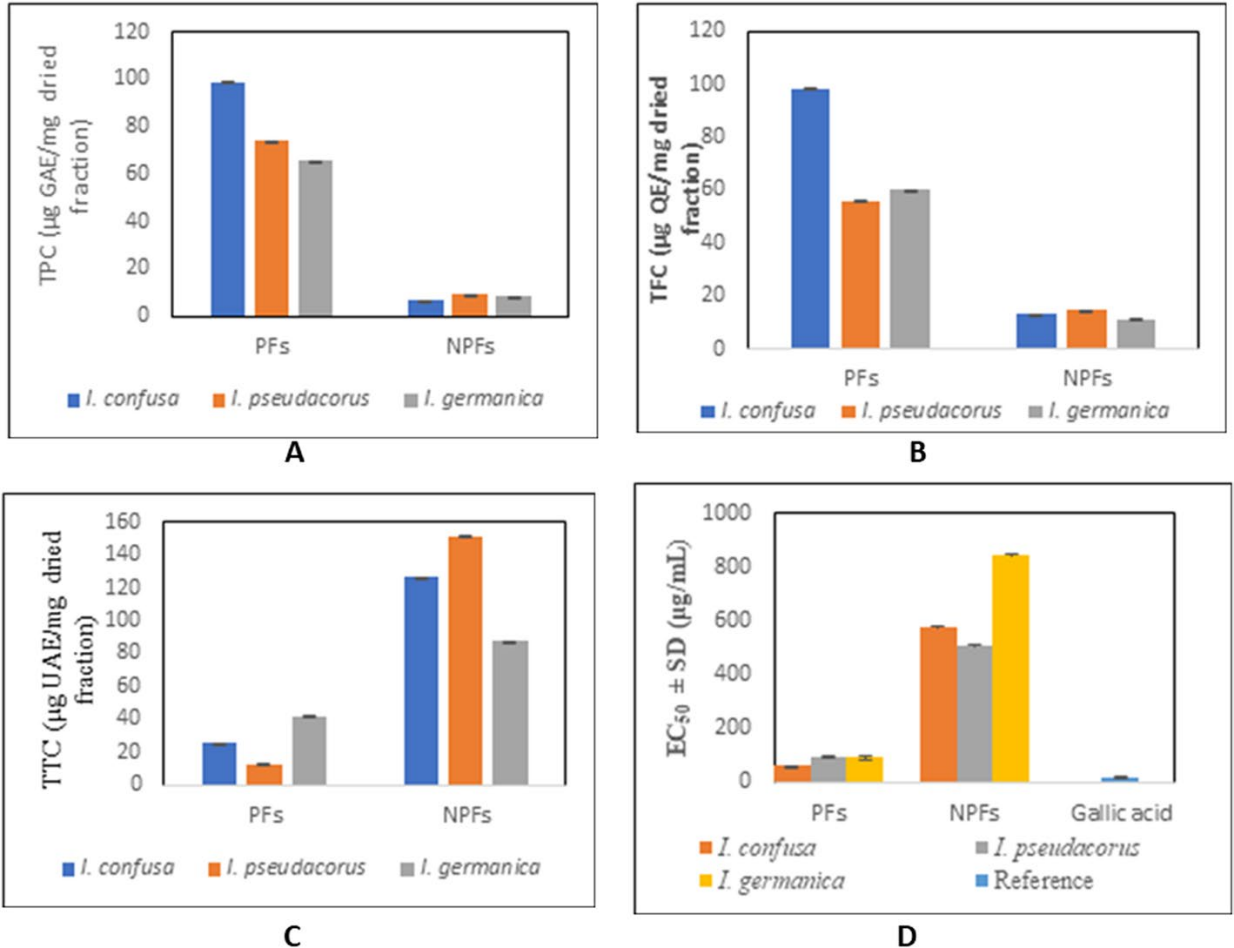
Quantification of total A) phenolic, B) flavonoid, and C) triterpene content calculated as gallic acid, quercetin, and ursolic acid equivalent respectively and D) DPPH antioxidant activity of polar and non-polar fractions of* I. pseudacorus, I. germanica and I. confusa* underground parts. GAE, gallic acid equivalent; TPC, total phenolic content; QE, quercetin equivalent; TFC, total flavonoid content; TTC, total triterpene content; UAE, ursolic acid equivalent; NPFs, non-polar fractions; PFs, polar fraction; EC_50_ = effective concentration of the sample required to scavenge 50% of the DPPH free radical

The three studied species NPFs generally showed higher triterpenoidal content while the PFs showed higher total phenolic and flavonoid contents (Fig. 5 A-C). The NPF of *I. pseudacorus* exhibited the highest TTC (151 ± 0.05 μg ursolic acid equivalent /mg dried fraction). On the other hand, *I. confusa* PF showed the highest content of TPC (99 ± 0.02 μg gallic acid equivalent /mg dried fraction) and TFC (98 ± 0.04 μg quercetin equivalent /mg dried fraction).

### Antioxidant activity

The EC_50_ of each sample against gallic acid is presented in Fig. 5D. All the studied PFs showed a stronger antioxidant activity than the corresponding NPFs. Among the PFs, *I. confusa* exhibited the highest DPPH scavenging activity with an EC_50_ of 41.68 ± 6.67 μg/mL.

### Drug likeness analysis

To explore the properties of the secondary metabolites correlated with NPF and PF of *Iris* species, Lipinski’s Rule of Five and Veber’s rules were predicted using the free accessible web server Swiss ADME (http://www.swissadme.ch/index.php). Results are tabulated in Table 2.

**Table 2:** Drug likeness prediction of the secondary metabolites strongly correlated with A) *I. confusa* NPF antiplasmodial and antileishmanial potentials and B) *I. pseudacorus* observed anti *Trypanosoma brucei* *rhodesiense* potential, using Lipinski and Veber filters

Molecule		Lipinski Filter			Veber Filter		
		Drug likeness	No. of violations	Violation	Drug likeness	No. of violations	Violation
(A)	Spirioiridoconfal C	yes	0	-	no	1	Rotatable bonds
	Oleic acid	yes	1	MLOGP	no	1	Rotatable bonds
	MGDG (18:3/18:3)	yes	1	MW	no	2	Rotatable bonds and TPSA
	Iridobelamal A	yes	1	MW	no	2	Rotatable bonds and TPSA
	TAG (18:2/18:2/18:3)	no	2	MW and MLOGP	no	1	Rotatable bonds
(B)	Cer (t18:0/α24:0)	no	2	MW and MLOGP	no	1	Rotatable bonds
	Cer (t18:1/α24:0)	no	2	MW and MLOGP	no	1	Rotatable bonds
	Chlorophyll b	yes	1	MW	no	1	Rotatable bonds
	Corosolic acid	yes	1	MLOGP	yes	0	-
	DGDG (18:3/18:3)	no	3	MW, #HBA, and #HBD	no	2	Rotatable bonds and TPSA
	Hydroxyoctadecadienoic acid	yes	0	-	no	1	Rotatable bonds
	Maslinic acid	yes	1	MLOGP	yes	0	-
	MGDG (18:2/18:2)	yes	1	MW	no	2	Rotatable bonds and TPSA
	Moreollic acid	yes	1	MW	yes	0	-
	PC (18:1/18:2)	yes	1	MW	no	1	Rotatable bonds
	PG (22:6/18:3)	no	2	MW and MLOGP	no	2	Rotatable bonds and TPSA
	Pheophytin a	no	2	MW and MLOGP	no	2	Rotatable bonds and TPSA
	Tumlosic acid	yes	1	MLOGP	yes	0	-

*HBA* Hydrogen bond acceptors, *HBD* Hydrogen bond donors, MLOGP Molecular logarithm of the octanol–water partition coefficient, *MW* molecular weight, *TPSA* Topological polar surface area

## Discussion

Cytotoxicity on host cells is a critical criterion for determining the selectivity of observed pharmacological effects and must always be considered in parallel. Although several cell types might conceivably be utilized for this purpose, MRC-5 cells were chosen due to their sensitivity and receptivity to a variety of parasites [11]. The CC_50_ of the tested fractions was also calculated against PMM, which are the host cells for *L. infantum* amastigotes, in order to assess the toxicity of the tested fractions on macrophages [36].

Herein, The NPFs showed significant antiplasmodial, antitrypanosomal and antileishmanial activities than the PFs. This could be due to the presence of several classes of biophytochemicals of well documented antiparasitic activities e.g. iridals [18], pentacyclic triterpenes [62] and phospholipids [63–65]. Interestingly, it has been shown in various parasitic infections that lipid synthesis increased dramatically in the infected cells to meet the parasite's need for new membranes as the parasite multiplied [65]. Thus, interfering with PL production with polar head analogues that compete or substitute for native polar head inclusion is fatal to certain parasites [66, 67]. It is worth noting that miltefosine, the reference medication for *L. infantum*, is a phospholipid analogue (hexadecylphosphocholine) [68]. The WHO Special Programme for Research & Training in Tropical Diseases (TDR) established an activity threshold as IC_50_ 0.2 µg /mL with SI > 20 for an antimalarial hit [69]. *I. confusa* NPF displayed the highest antiplasmodial activity with high selectivity index. In addition, it was the only fraction exerting an inhibitory activity against *L. infantum* with no cytotoxicity. Five metabolites in* I. confusa* NPF were strongly correlated with its high antiplasmodial and antileshmanial potentials; glycolipids [MGDG (18:3/18:3)], triacyl glycerols [TAG (18:2/18:2/18:3), iridals (spirioiridoconfal C and, (iso)iridobelamal) and fatty acid (oleic acid). These phytochemical classes are well reputed for their antiplasmodial and antileishmanial activities. Iridals were previously reported to exhibit antiplasmodial activity [19]. In addition, fatty acids showed inhibitory action against the fatty acid biosynthetic machinery of the parasite *P. falciparum* which could be considered as a likely strategy to combat the parasite [70, 71]. Glycolipids were previously reported to exhibit antiplasmodial and antileishmanial activities [72]. Triacylglycerols exhibited promising antileishmanial activities [73].

*I. pseudacorus* NPF showed the higheset activity against *T. b. rhodesiense.* Thirteen metabolites were correlated to this activity including triterpene acids (tumlosic acid and corosolic/maslinic acid), certain ceramides [cer (t18:1/*α*24:0) and ceramide (t 18:0/*α*24:0), caged xanthone (moreollic acid), chlorophyll derivatives (pheophytin a and chlorophyll b), phosphatidylglycerol [PG (22:6/18:3)], glycolipids [DGDG (18:3/18:3), and MGDG 18:2/18:2)] triterpenoid irridal iso(iridobelamal), fatty acid (hydroxyoctadecadienoic acid), phosphatidylcholine (PC 18:1/18:2). This could be justified by the previously reported antitrypanosomal activity of many of those compounds as corosolic and maslinic acids [74], xanthones [75], fatty acids [76], iridals [18], ceramides [77] and pheophytin a [78].

The 2,2-diphenyl-1-picrylhydrazyl radical (DPPH) *in-vitro* model was used in this study to assess the antioxidant capacity of the PFs and NPFs fractions of the three studied species.

The observed higher triterpenoidal content of the NPFs matched with the documented antiprotozoal activity of triterpenes [79–81]. The higher DPPH scavenging activity of the PFs than the NFs was in accordance with their higher TPC and TFC [82]. This was justified by the highest scavenging activity of *I. confusa* PF that showed the highest TPC and TFC. However, the observed antioxidant potential of the NPFs could be attributed to their xanthones and triterpenoid contents [83, 84].

A future study is required to isolate the metabolites of the active fraction(s) responsible for the antiparasitic activity. Future studies concerning the validation of this in vitro results by detailed in vivo, bioavailability, and safety studies are highly recommended.

## Conclusion

For the first time, a comparative evaluation of the antiplasmodial, antileishmanial and antitrypanosolmal potentials of *I. pseudacorus, I. germanica* and *I. confusa* in relation to their metabolic profile was performed. Herein, the antiplasmodial potential of *I. confusa* NPF was highlighted in a first record. Our future perspective is fractionation of *I. confusa* NPF to less complex fraction or purified compounds with the aim of discovering new antiplasmodial hits that meet the requirements of WHO.

### Supplementary Information

**Additional file 1**

## Data Availability

All data generated or analyzed during this study are included in this published article and its supplementary information file.

## Abbreviations

CC_50_: 50% Cytotoxic concentration
CPRG: Chlorophenol red *β*-D-galactopyranoside
DF: Dried fraction
DMSO: Dimethylsulfoxide
FCSi: Fetal calf serum
GAE: Gallic acid equivalent
HAT: Human African Trypanosomiasis
HMI-9: Hirumi-9
IC_50_: Inhibitory concentration 50%
MEM: Minimal essential medium
MRC-5: Human embryonic lung fibroblasts
MTBE: Methyl tert-butyl ether
MTT: 3-[4,5-Dimethylthiazol-2-yl]- 2,5-diphenyltetrazolium bromide
NBT: Nitro blue tetrazolium
NPF: Non-polar fraction
PF: Polar fraction
PES: Phenazine ethosulfate
PMM: Primary peritoneal murine macrophages
QE: Quercetin equivalent
SI: Selectivity index
UAE: Ursolic acid equivalent
UPLC-HRMS/MS: Ultra-performance liquid chromatography coupled to high-resolution Tandem Mass Spectrometry
WHO: The world health organisation
