# Supplementary material for: Correlation between secondary metabolites of Iris confusa Sealy and Iris pseudacorus L. and their newly explored antiprotozoal potentials
Source: BMC Complement Med Ther. 2023 Dec 16;23:465. doi: 10.1186/s12906-023-04294-0 (PMC10725014; doi:10.1186/s12906-023-04294-0)
Supplement: Supplementary file 1 — Additional file 1 [file 12906_2023_4294_MOESM1_ESM.pdf]

Supplementary Material

Correlation between secondary metabolites of *Iris confusa* Sealy and *Iris pseudacorus* L. and their newly explored antiprotozoal potentials

Passent M. Abdel-Baki[1*], Moshera M. El-Sherei[1], Amal E. Khaleel[1], Essam Abdel-Sattar[1] and Mohamed A. Salem[2], , Mona M. Okba[1]

[1]*Department of Pharmacognosy, Faculty of Pharmacy, Cairo University, Cairo, Kasr-El-Ainy Street, 11562, Cairo, Egypt.*
[2]*Department of Pharmacognosy, Faculty of Pharmacy, Menoufia University, Gamal Abd El Nasr st., Shibin Elkom, 32511, Menoufia, Egypt.*

*Corresponding author: passent.mohamed@pharma.cu.edu.eg, 01111804949

Supplementary figures

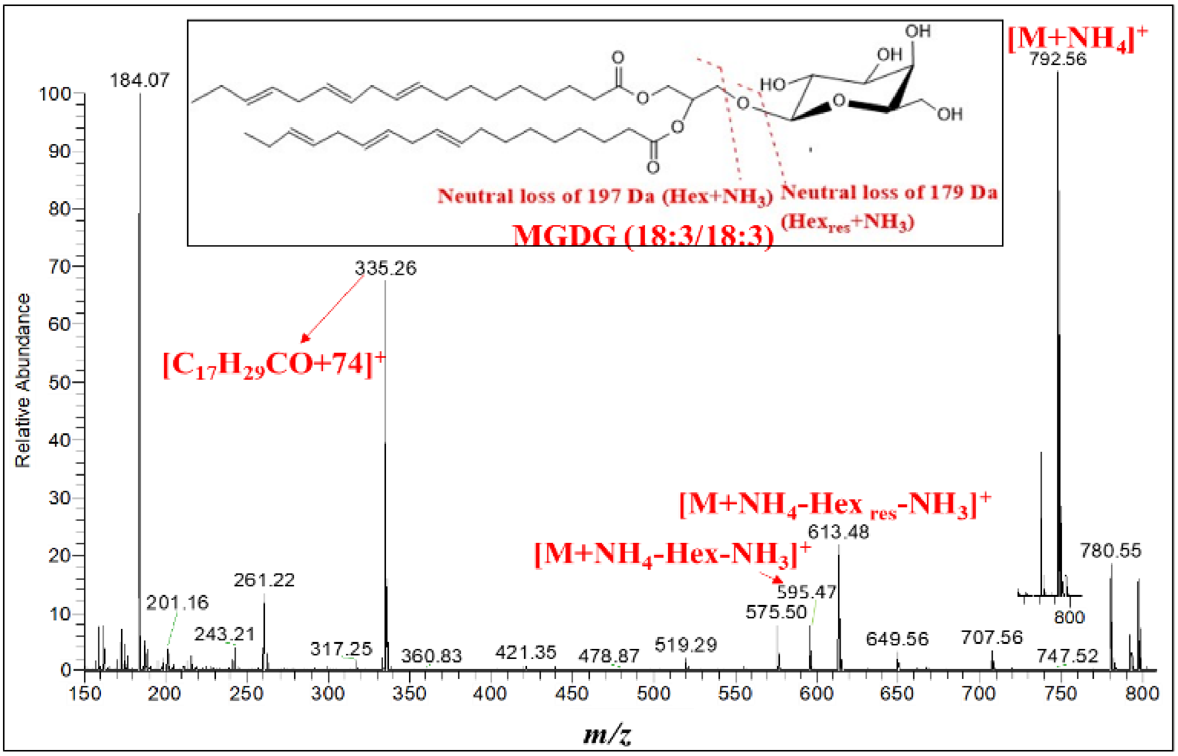

A

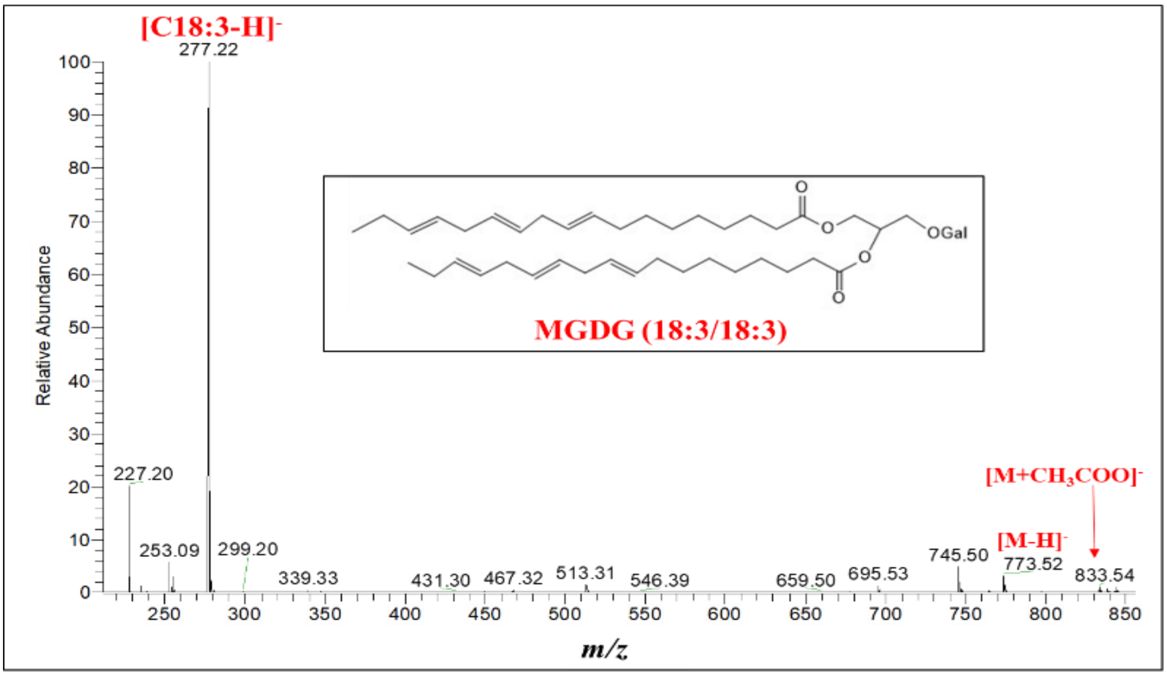

B

Fig. S1. Ms/Ms chromatograms of metabolite no. 1 identified in *I. confusa* non-polar fraction A) positive, and B) negative mode [1].

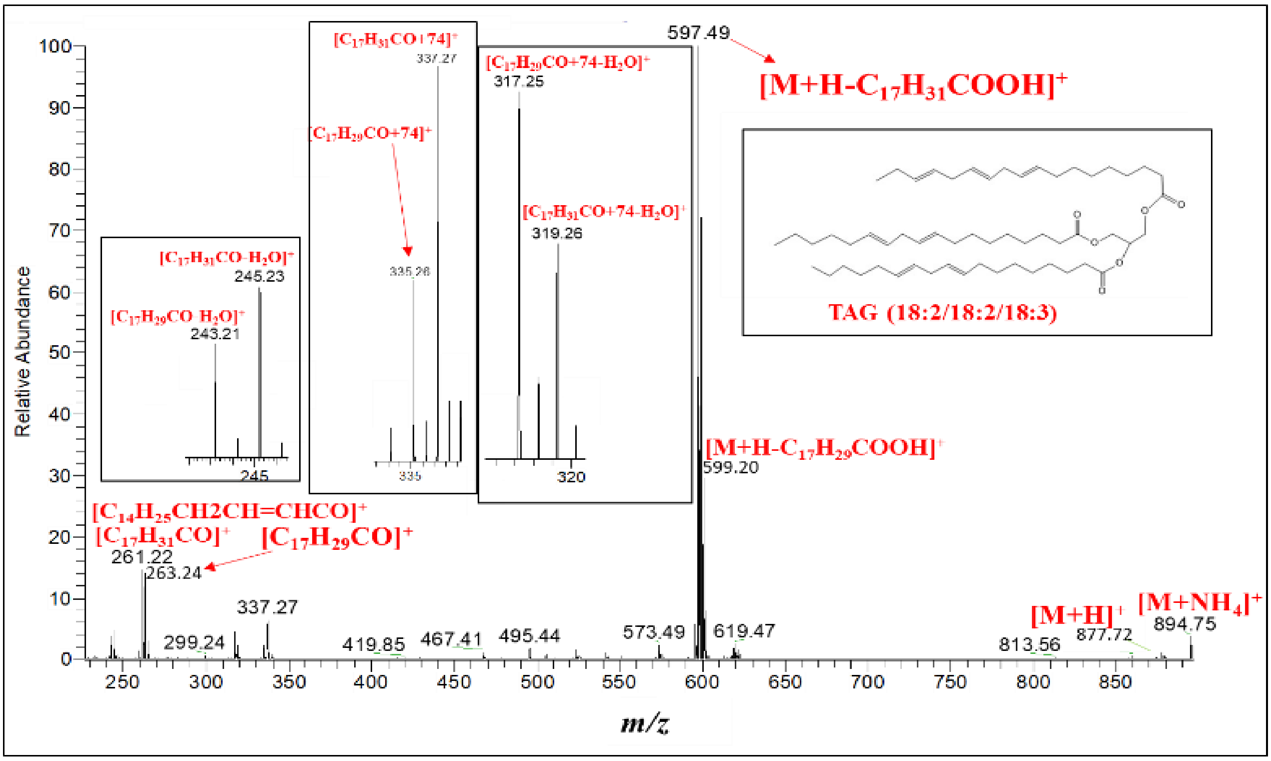

Fig. S2. Ms/Ms chromatograms of metabolite no. 2 identified in *I. confusa* non-polar fraction in positive mode [1]..

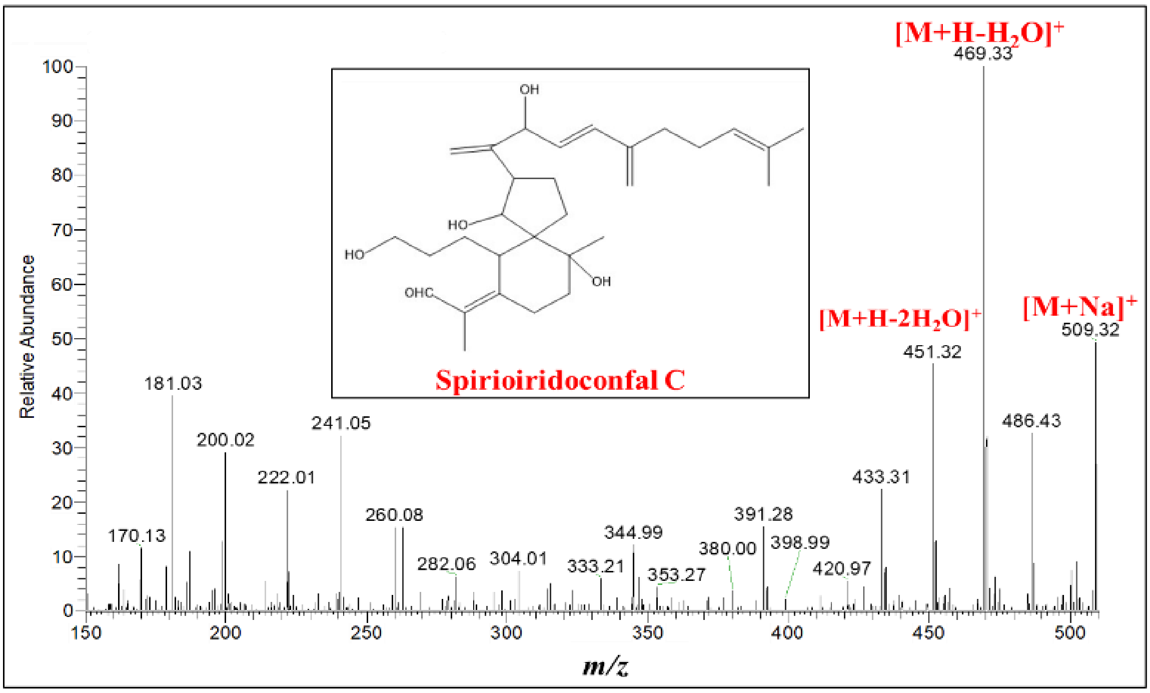

A

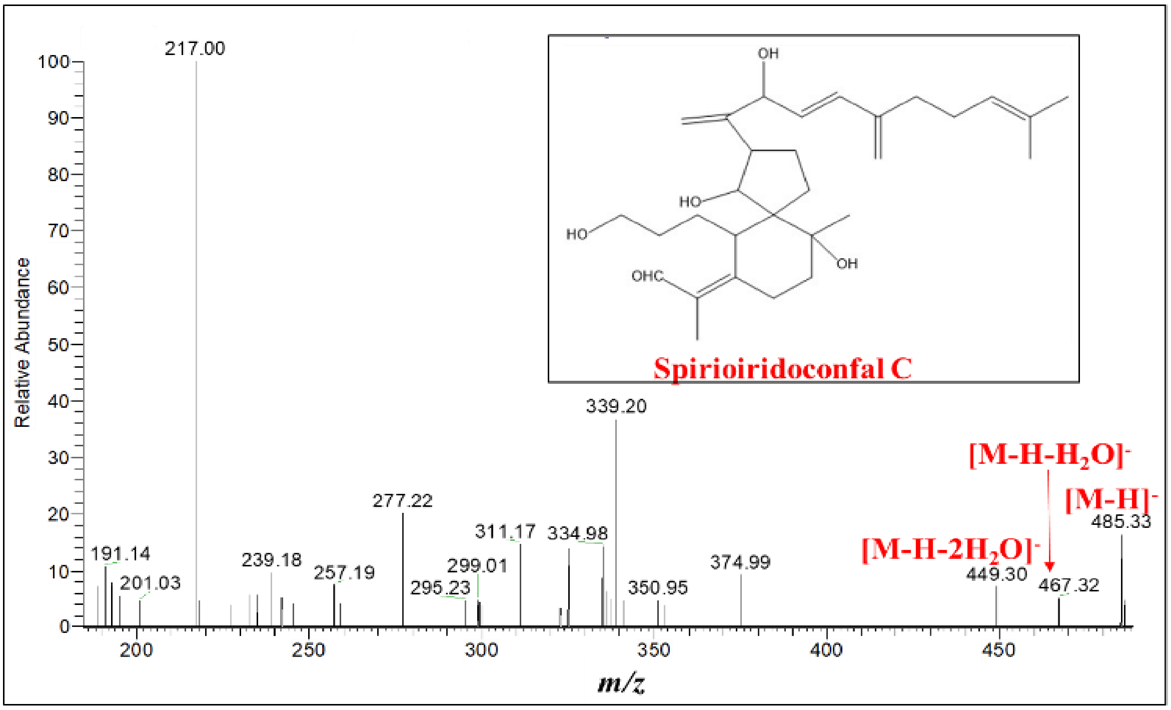

B

Fig. S3. Ms/Ms chromatograms of metabolite no. 3 identified in *I. confusa* non-polar fraction A) positive, and B) negative mode [1]..

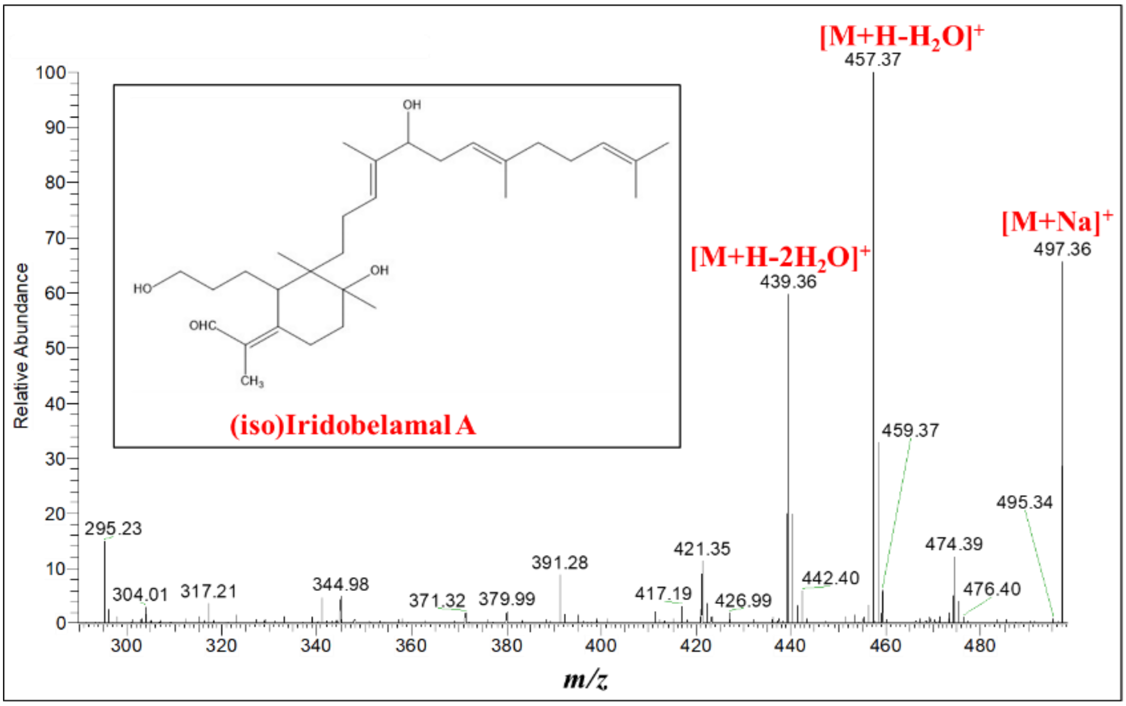

A

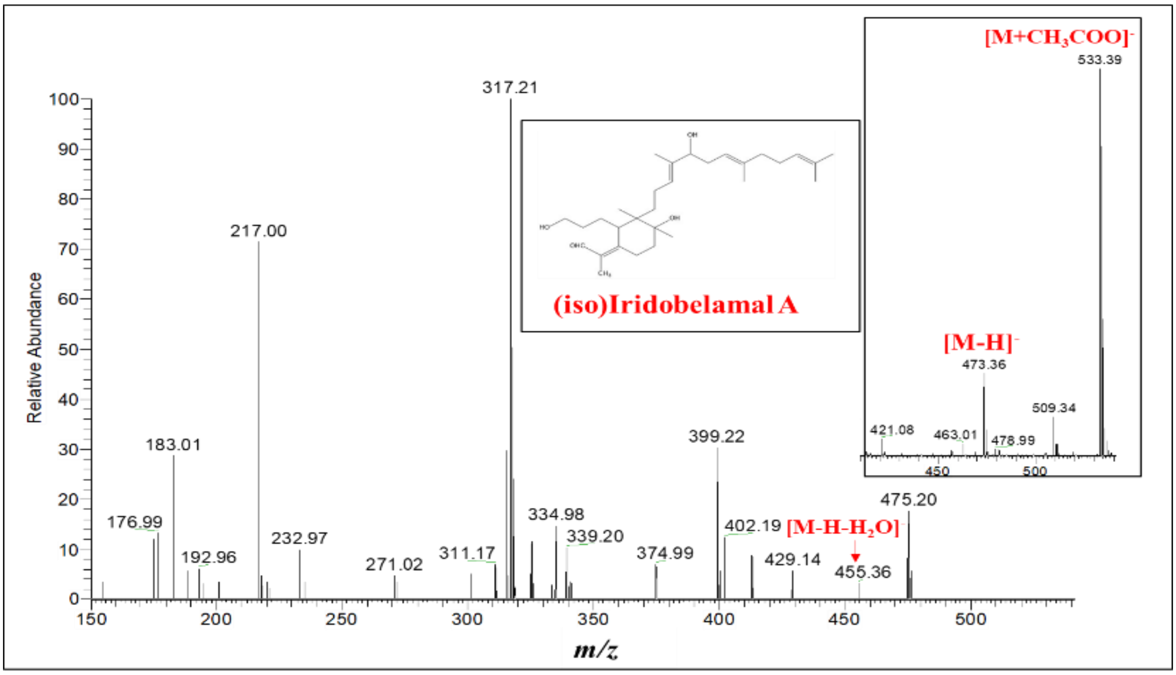

B

Fig. S4. Ms/Ms chromatograms of metabolite no. 4 identified in *I. confusa* non-polar fraction A) positive, and B) negative mode [1]..

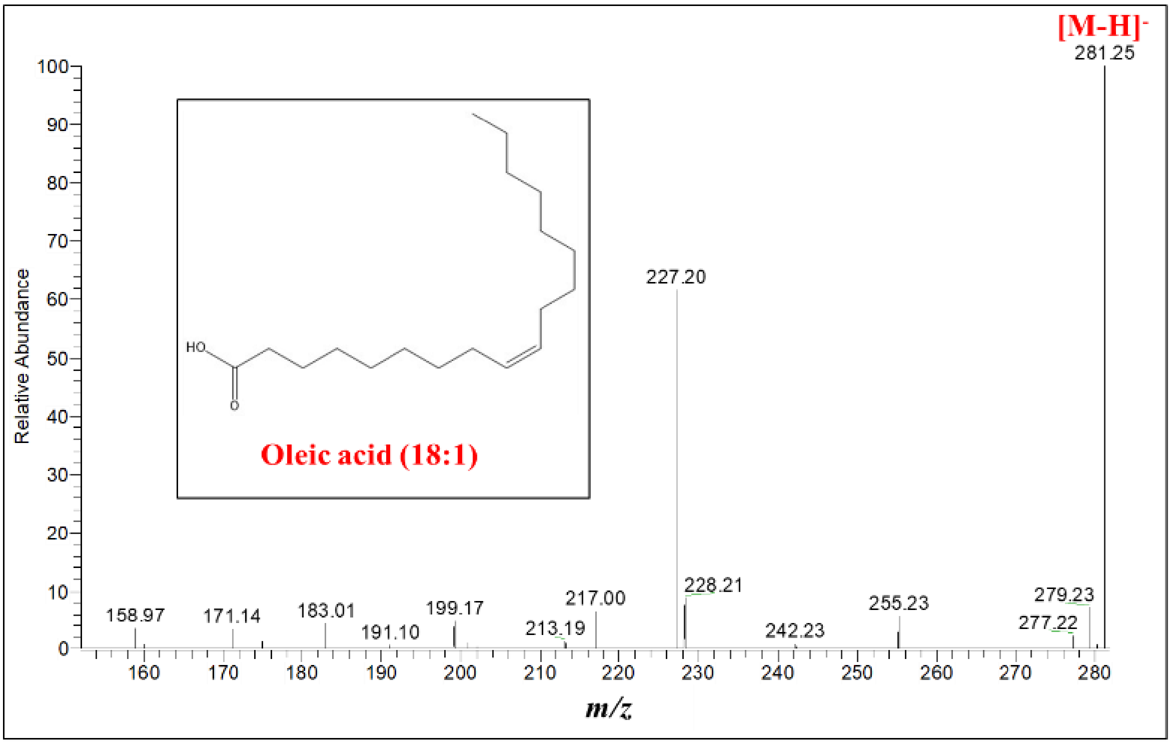

Fig. S5. Ms/Ms chromatograms of metabolite no. 5 identified in *I. confusa* non-polar fraction in positive mode [1]..

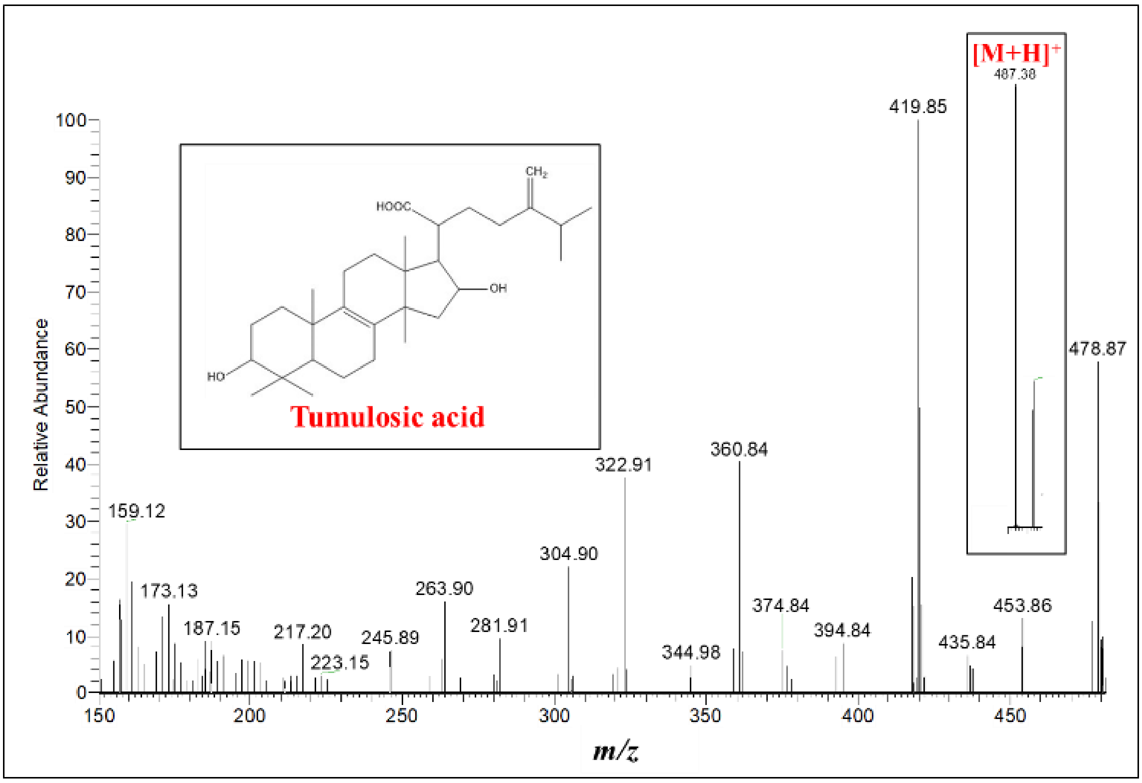

A

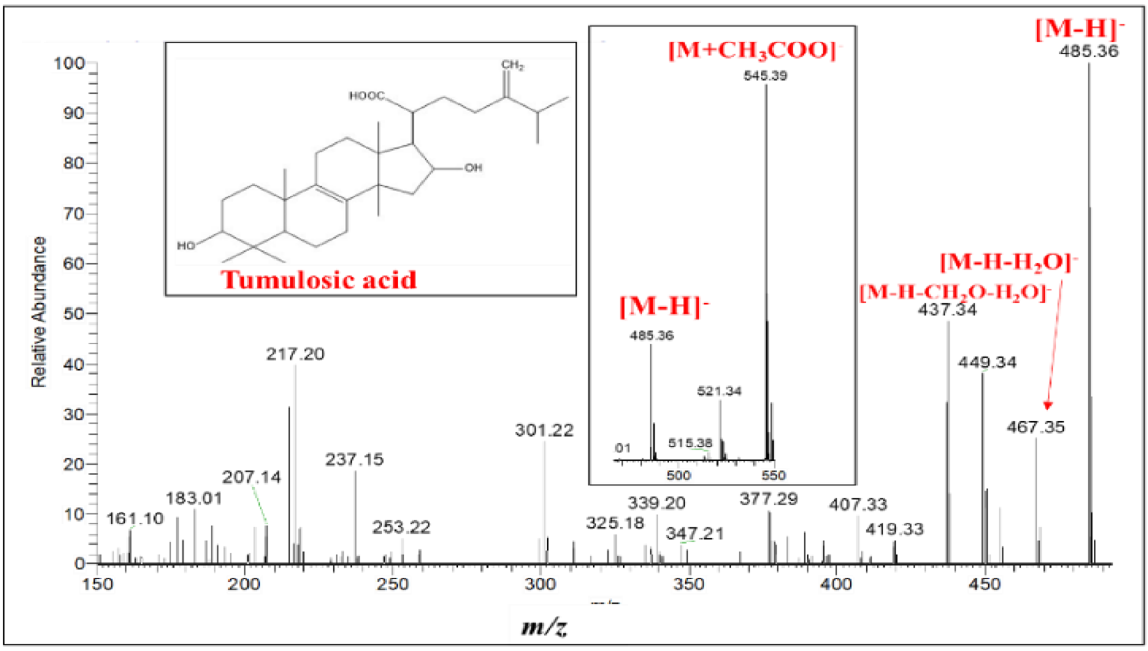

B

Fig. S6. Ms/Ms chromatograms of metabolite no. 6 identified in *I. pseudacorus* non-polar fraction A) positive, and B) negative mode [1]..

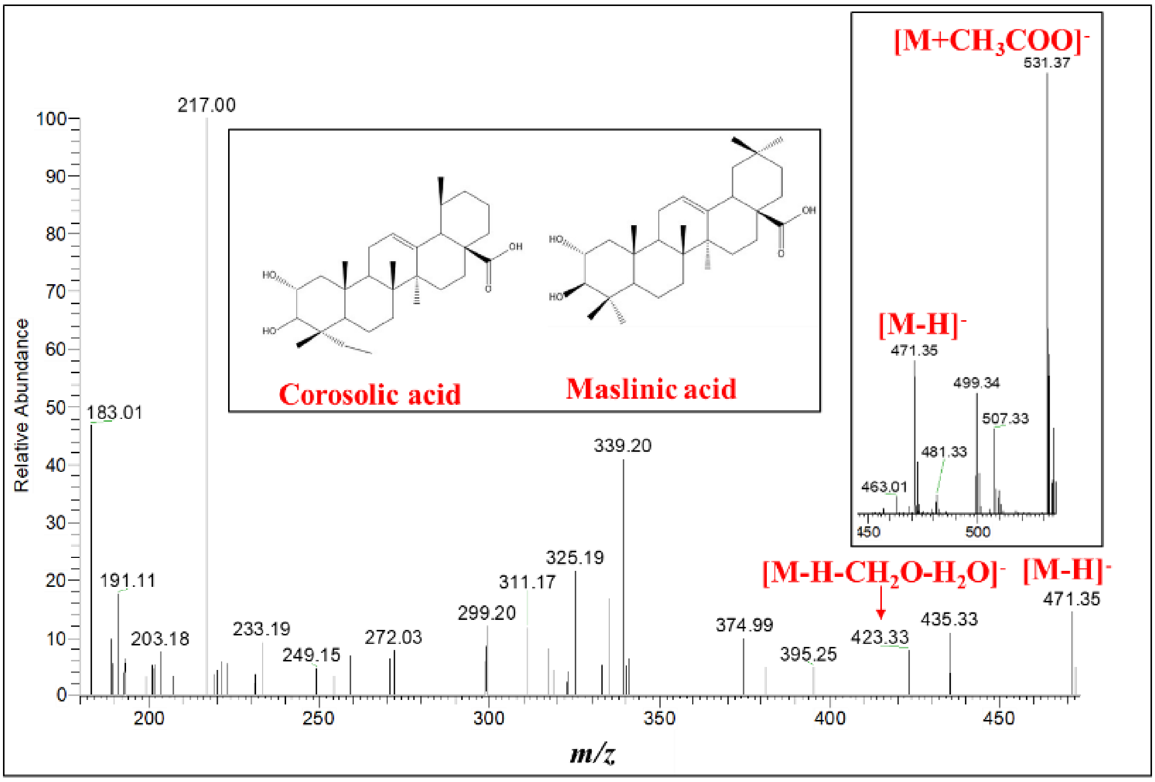

Fig. S7. Ms/Ms chromatograms of metabolite no. 7 identified in *I. pseudacorus* non-polar fraction in negative mode [1]..

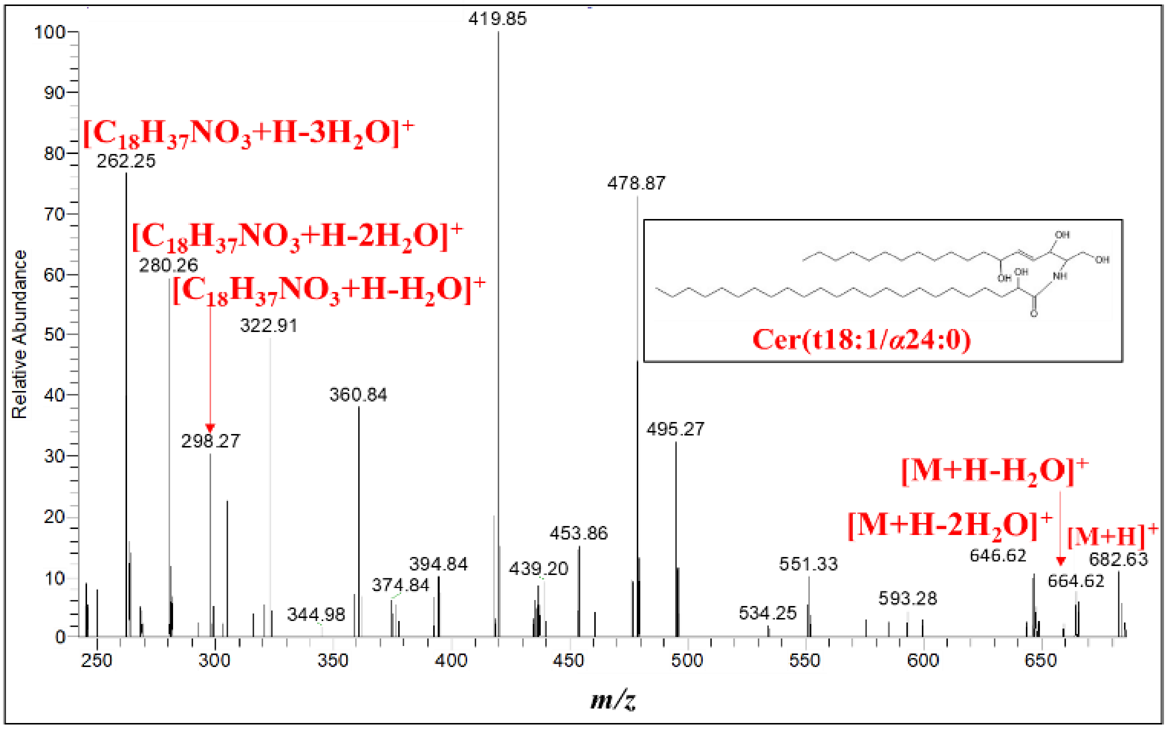

A

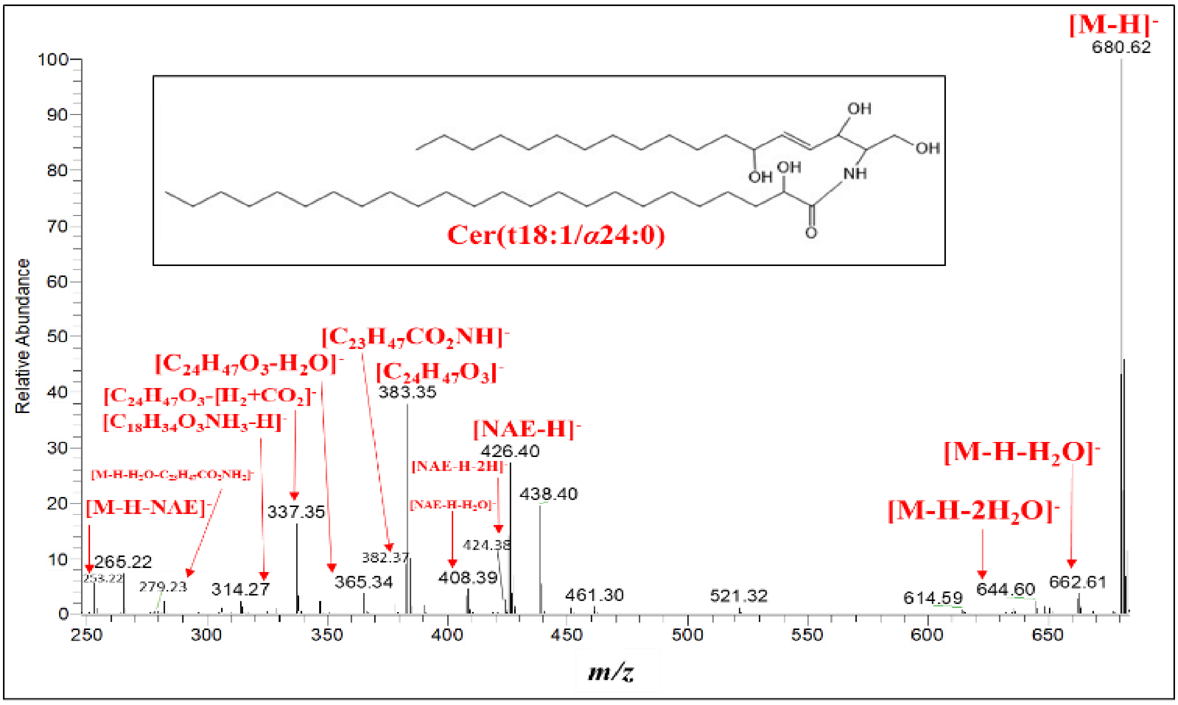

B

Fig. S8. Ms/Ms chromatograms of metabolite no. 8 identified in *I. pseudacorus* non-polar fraction A) positive, and B) negative mode [1]..

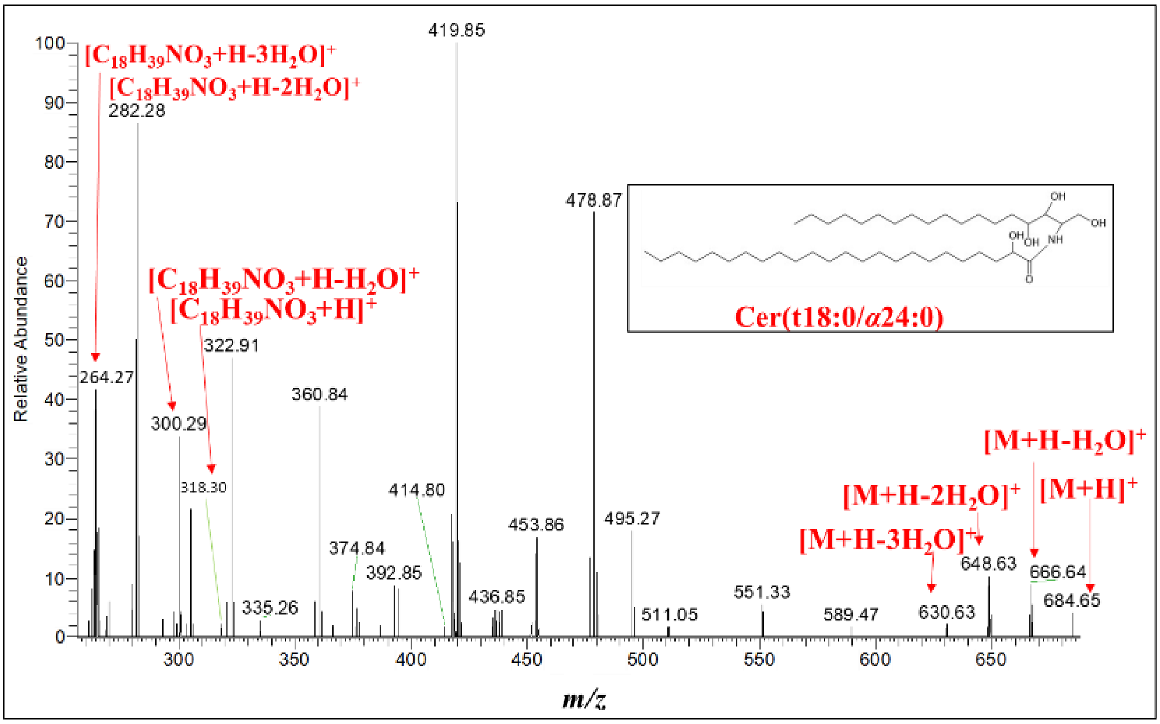

A

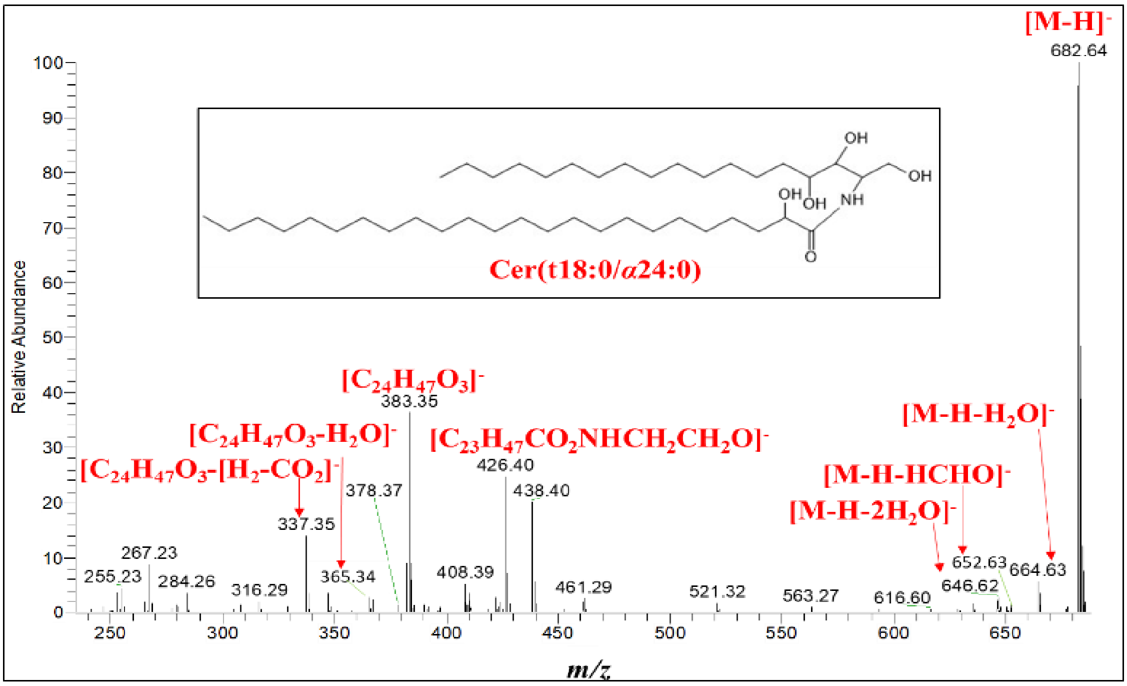

B

Fig. S9. Ms/Ms chromatograms of metabolite no. 9 identified in *I. pseudacorus* non-polar fraction A) positive, and B) negative mode [1]..

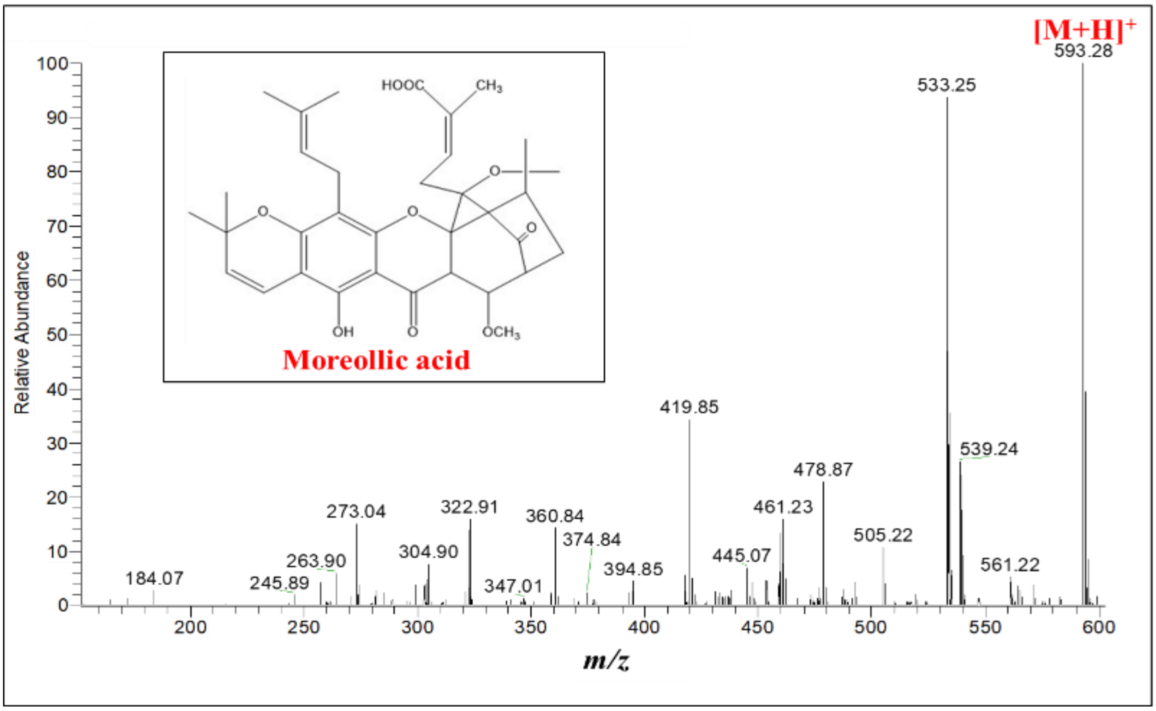

Fig. S10. Ms/Ms chromatograms of metabolite no. 10 identified in *I. pseudacorus* non-polar fraction in positive mode [1]..

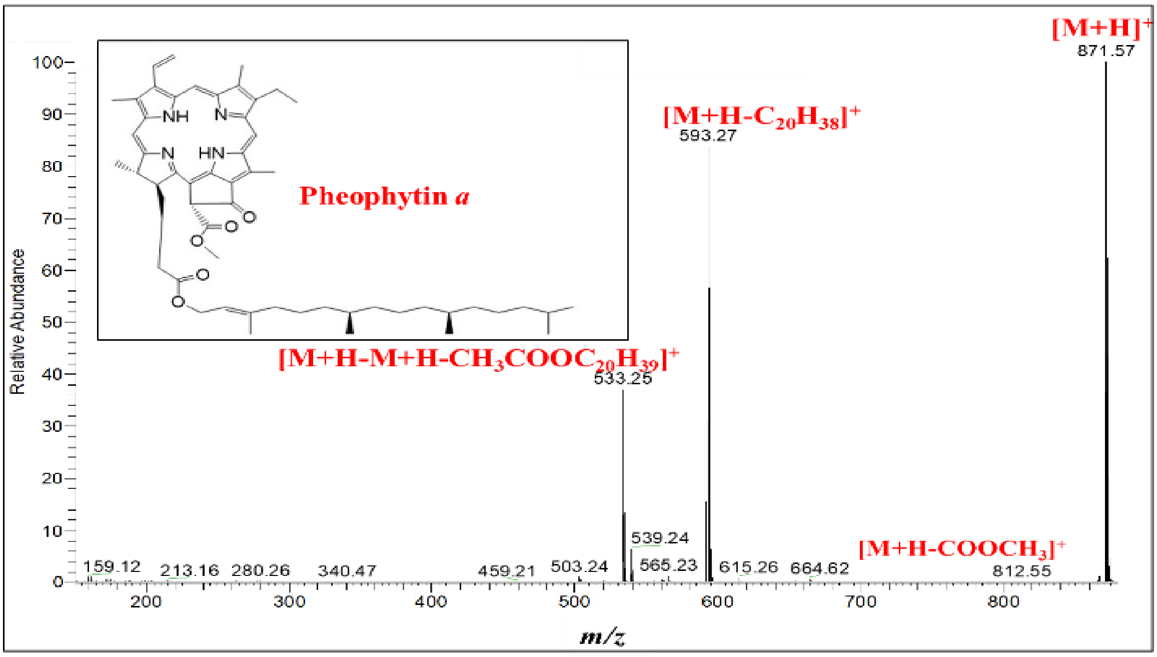

Fig. S11. Ms/Ms chromatograms of metabolite no. 11 identified in *I. pseudacorus* non-polar fraction in positive mode [1]..

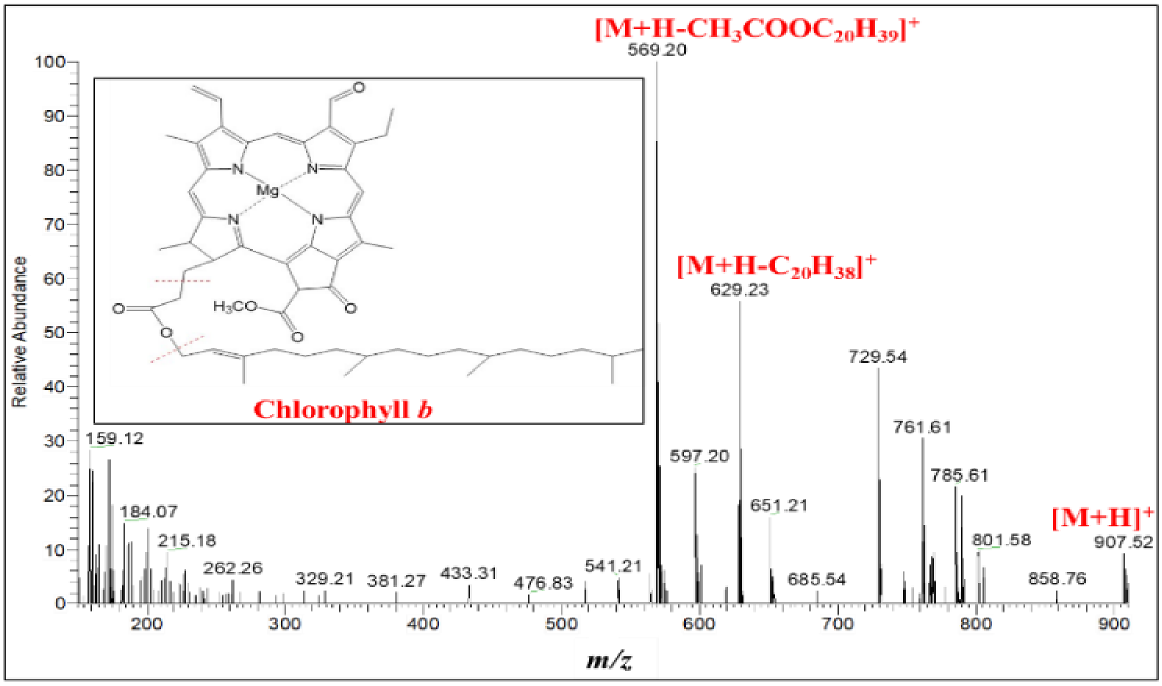

A

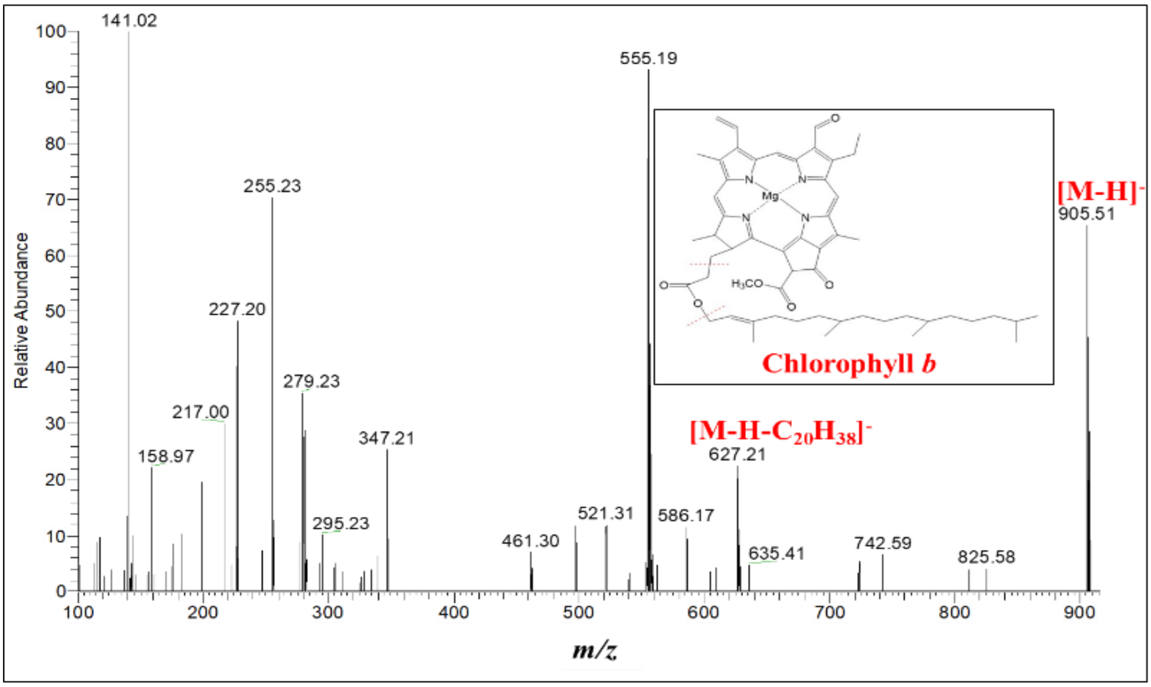

B

Fig. S12. Ms/Ms chromatograms of metabolite no. 12 identified in *I. pseudacorus* non-polar fraction A) positive, and B) negative mode [1]..

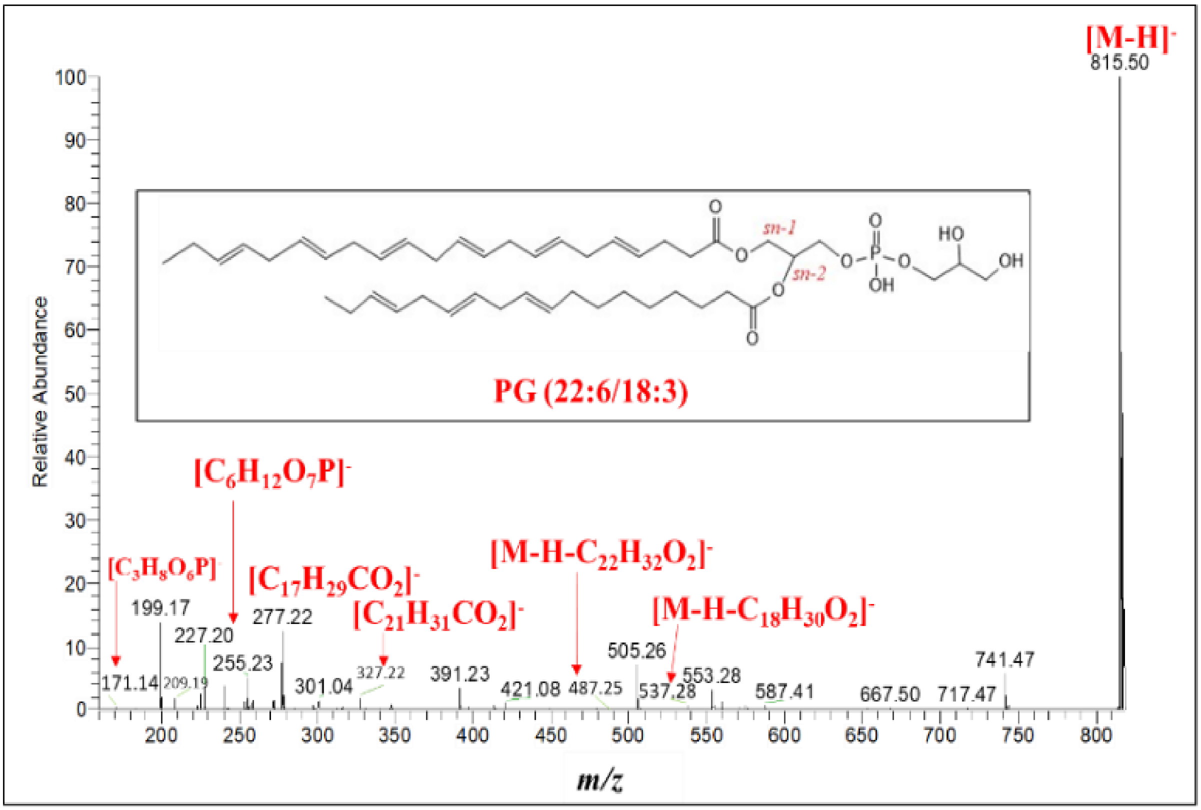

Fig. S13. Ms/Ms chromatograms of metabolite no. 13 identified in *I. pseudacorus* non-polar fraction in negative mode [1]..

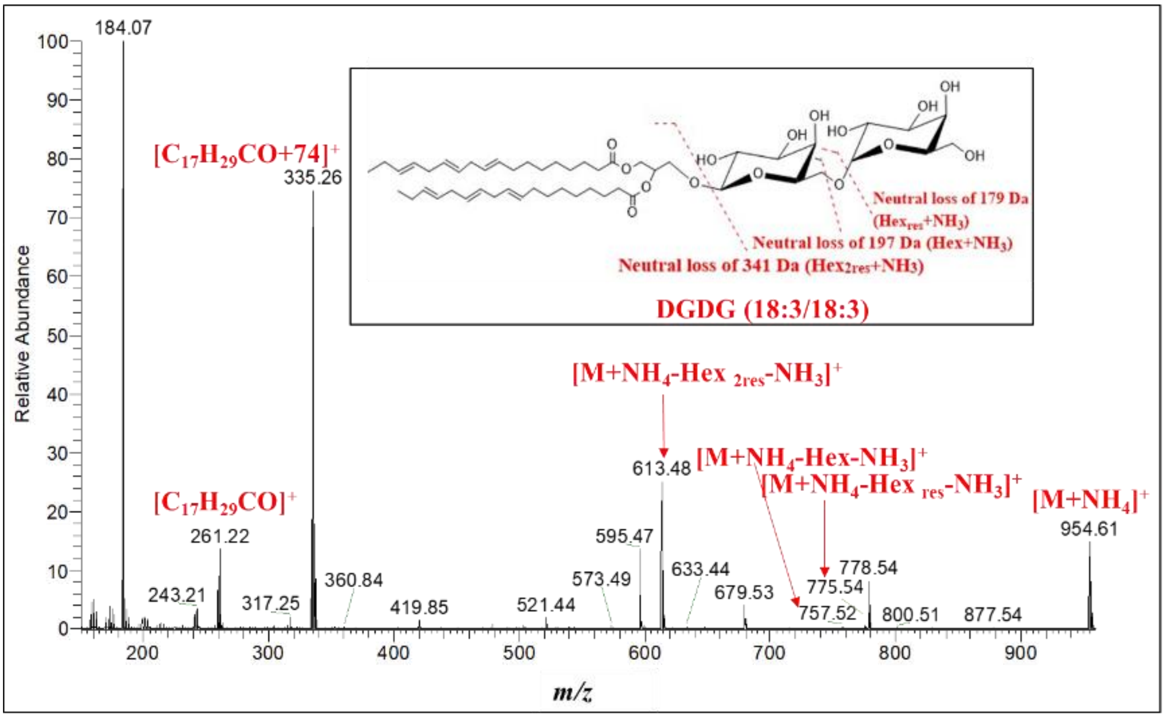

A

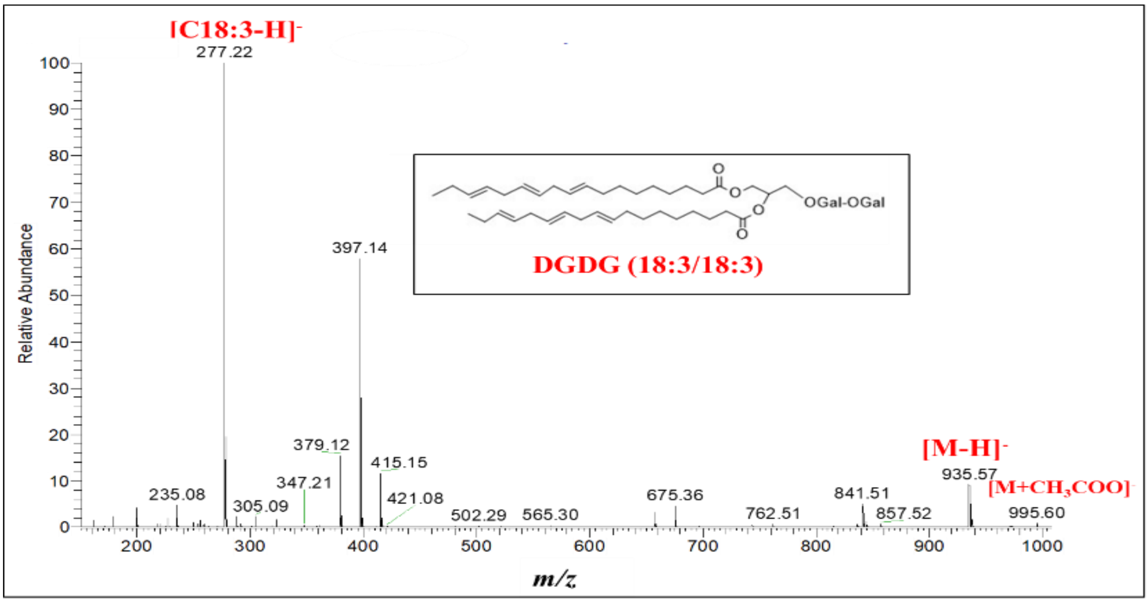

B

Fig. S14. Ms/Ms chromatograms of metabolite no. 14 identified in *I. pseudacorus* non-polar fraction A) positive, and B) negative mode [1]..

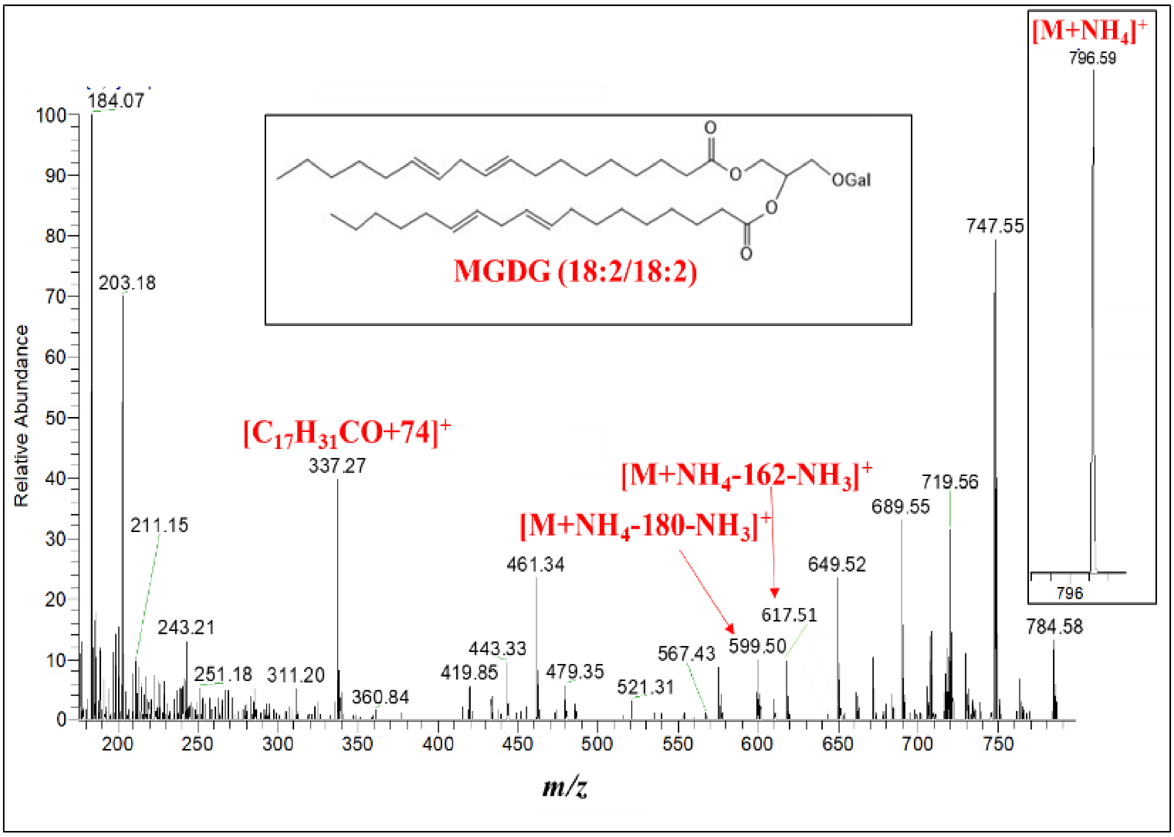

A

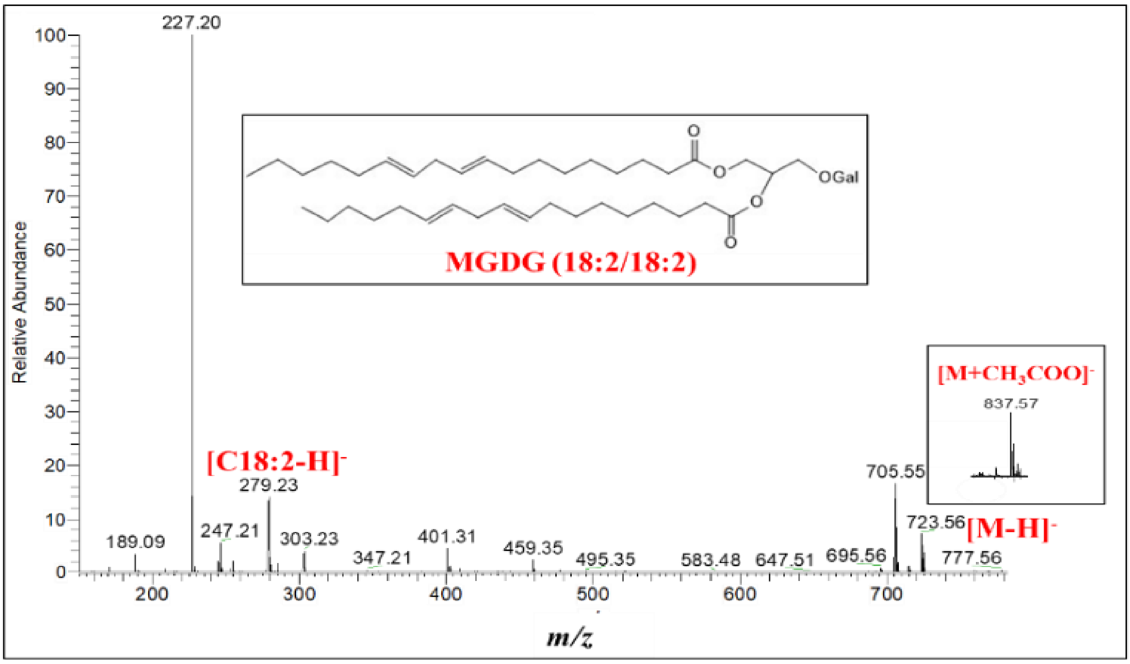

B

Fig. S15. Ms/Ms chromatograms of metabolite no. 15 identified in *I. pseudacorus* non-polar fraction A) positive, and B) negative mode [1]..

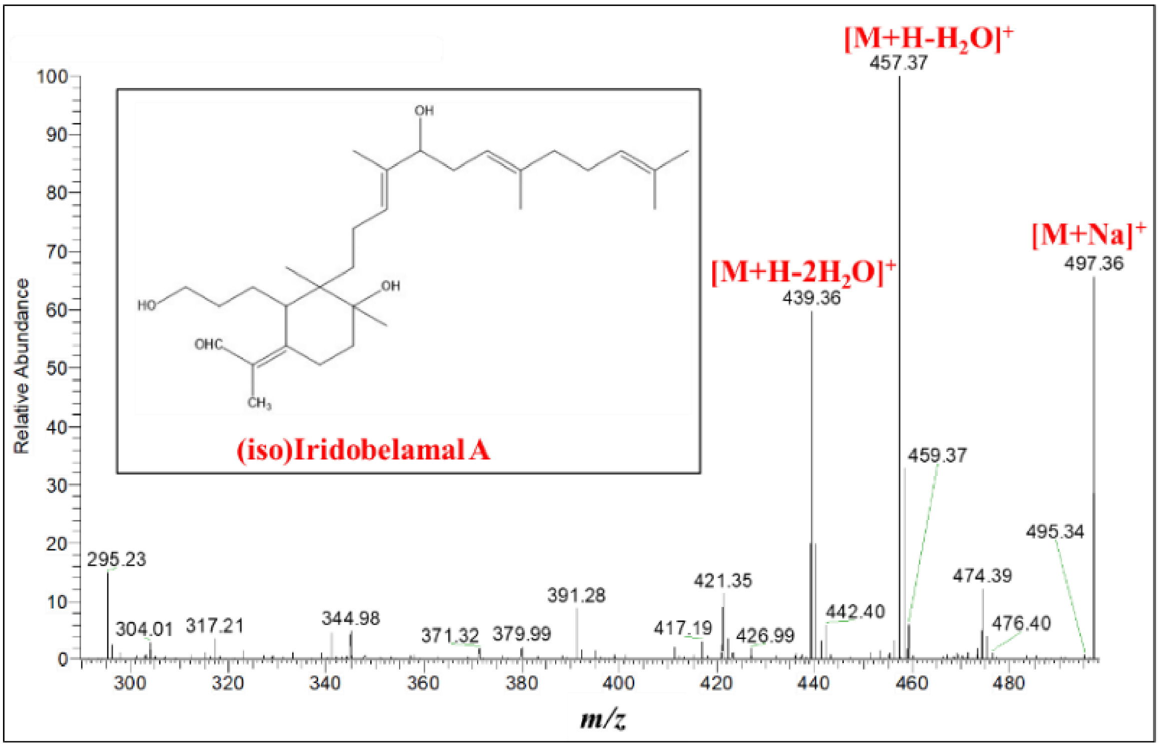

A

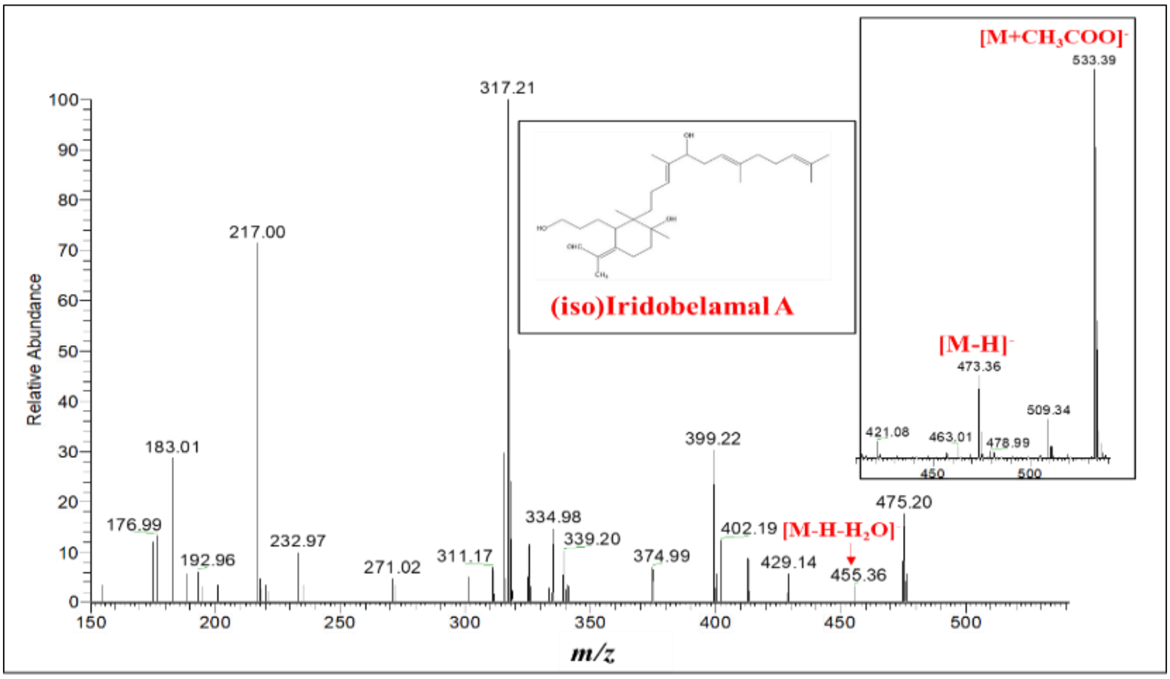

B

Fig. S16. Ms/Ms chromatograms of metabolite no. 16 identified in *I. pseudacorus* non-polar fraction A) positive, and B) negative mode [1]..

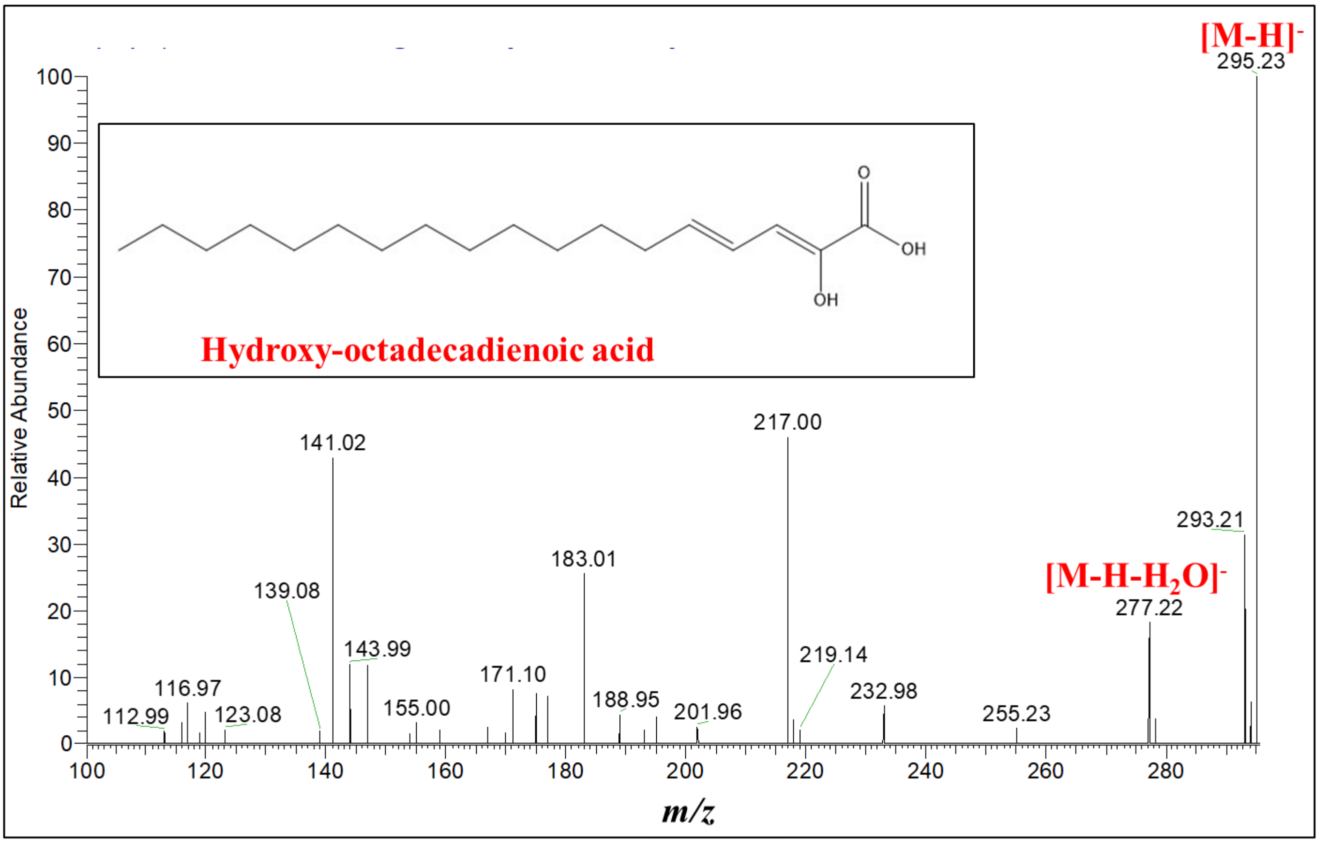

Fig. S17. Ms/Ms chromatograms of metabolite no. 17 identified in *I. pseudacorus* non-polar fraction in negative mode [1]..

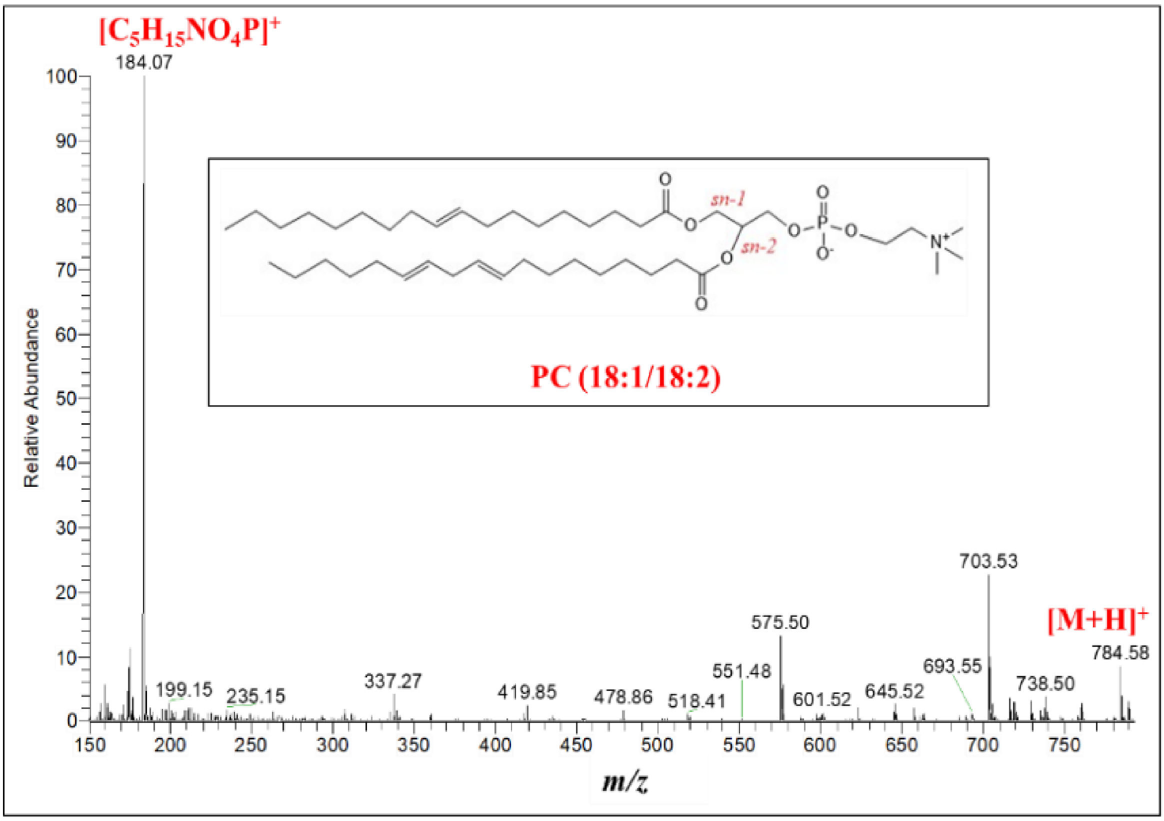

A

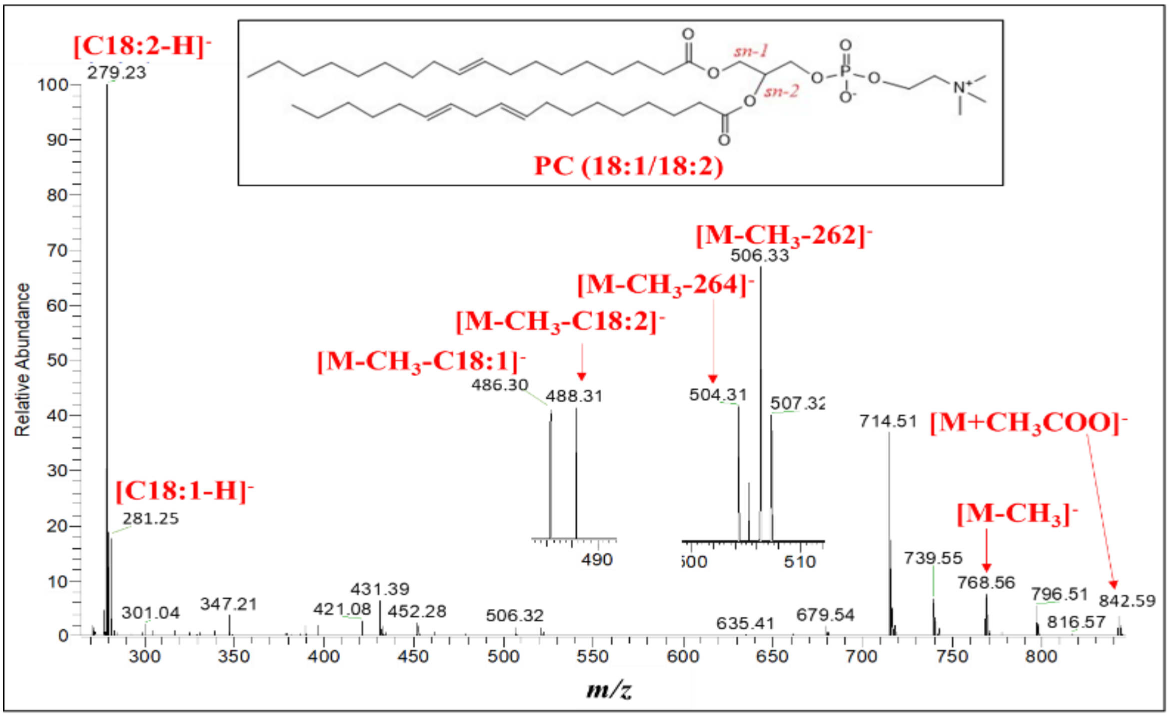

B

Fig. S18. Ms/Ms chromatograms of metabolite no. 18 identified in *I. pseudacorus* non-polar fraction A) positive, and B) negative mode [1]..

Reference

1. Okba MM, Baki PMA, Abu-Elghait M et al. UPLC-ESI-MS/MS profiling of the underground parts of common *Iris* species in relation to their anti-virulence activities against *Staphylococcus aureus*. J Ethnopharmacol. 2022; 282: 114658. DOI: https://doi.org/10.1016/j.jep.2021.114658
